# PRIM1 deficiency causes a distinctive primordial dwarfism syndrome

**DOI:** 10.1101/gad.340190.120

**Published:** 2020-11-01

**Authors:** David A. Parry, Lukas Tamayo-Orrego, Paula Carroll, Joseph A. Marsh, Philip Greene, Olga Murina, Carolina Uggenti, Andrea Leitch, The Scottish Genomes Partnership, Rita Káposzta, Gabriella Merő, Andrea Nagy, Brigitta Orlik, Balázs Kovács-Pászthy, Alan J. Quigley, Magdolna Riszter, Julia Rankin, Martin A.M. Reijns, Katalin Szakszon, Andrew P. Jackson

**Affiliations:** 1 MRC Human Genetics Unit, MRC Institute of Genetics and Molecular Medicine, the University of Edinburgh, Edinburgh EH4 2XU, United Kingdom;; 2 Centre for Genomic and Experimental Medicine, MRC Institute of Genetics and Molecular Medicine, the University of Edinburgh, Edinburgh EH4 2XU, United Kingdom;; 3 Institute of Pediatrics, Faculty of Medicine, University of Debrecen, Debrecen H-4032, Hungary;; 4 Institute of Pathology, Faculty of Medicine, University of Debrecen, Debrecen H-4032, Hungary;; 5 Department of Radiology, Royal Hospital for Sick Children, Edinburgh EH9 1LF, United Kingdom;; 6 Department Clinical Genetics, Royal Devon and Exeter NHS Foundation Trust, Exeter EX1 2ED, United Kingdom; 9 Centre for Genomic and Experimental Medicine, MRC Institute of Genetics and Molecular Medicine, the University of Edinburgh, Edinburgh EH4 2XU, United Kingdom; 10 Wolfson Wohl Cancer Research Centre, Institute of Cancer Sciences, University of Glasgow, Glasgow G61 1QH, United Kingdom; 11 University of Aberdeen, Aberdeen AB24 3FX, United Kingdom; 12 MRC Human Genetics Unit, MRC Institute of Genetics and Molecular Medicine, the University of Edinburgh, Edinburgh EH4 2XU, United Kingdom; 13 Department of Medical Genetics, Ashgrove House, NHS Grampian, Aberdeen AB25 2ZA, United Kingdom; 14 National Services Division, NHS National Services Scotland, Edinburgh EH12 9EB, United Kingdom; 15 Edinburgh Genomics Clinical Division, the Roslin Institute, University of Edinburgh, Edinburgh EH25 9RG, United Kingdom; 16 West of Scotland Genetic Services, Queen Elizabeth University Hospital, Glasgow G51 4TF, United Kingdom

**Keywords:** DNA replication, genome stability, growth disorders, human genetics, rare disease

## Abstract

DNA replication is fundamental for cell proliferation in all organisms. Nonetheless, components of the replisome have been implicated in human disease, and here we report *PRIM1* encoding the catalytic subunit of DNA primase as a novel disease gene. Using a variant classification agnostic approach, biallelic mutations in PRIM1 were identified in five individuals. PRIM1 protein levels were markedly reduced in patient cells, accompanied by replication fork asymmetry, increased interorigin distances, replication stress, and prolonged S-phase duration. Consequently, cell proliferation was markedly impaired, explaining the patients’ extreme growth failure. Notably, phenotypic features distinct from those previously reported with DNA polymerase genes were evident, highlighting differing developmental requirements for this core replisome component that warrant future investigation.

## Abstract (precis)

In this study, Parry et al. report PRIM1, encoding the catalytic subunit of DNA primase, as a novel disease gene. Using a variant classification agnostic approach, biallelic mutations in PRIM1 were identified in five individuals and phenotypic features distinct from those previously reported with DNA polymerase genes were evident, highlighting differing developmental requirements for this core replisome component.

Microcephalic primordial dwarfism (MPD) disorders constitute several Mendelian syndromes characterized by intrauterine growth retardation, short stature, and microcephaly (Klingseisen and Jackson 2011). Previously, partial loss-of-function mutations have been identified in genes encoding fundamental components of the cell cycle machinery, suggesting a model in which disrupted cell proliferation during development results in “hypocellular” forms of dwarfism (Klingseisen and Jackson 2011).

Perturbation of components of the DNA replication machinery has emerged as a common cause of MPD, involving several stages of the replication process. In the G1-phase of the cell cycle the prereplication complex acts to license replication origins, and subsequently the replication preinitiation complex is formed, with the full replisome assembled at S-phase entry, to initiate DNA replication effected by three DNA polymerases: α, δ, and ε (Pol α, δ, and ε). Biallelic mutations in components of the prereplication complex were first identified, with *ORC1*, *ORC4*, *ORC6*, *CDT1*, and *CDC6*, causing Meier-Gorlin syndrome (MGS) (Bicknell et al. 2011a,b; Guernsey et al. 2011), a disorder defined by a triad of microtia, patella hypoplasia, and growth restriction. Heterozygous stabilizing mutations in *GMNN* and recessive variants in *CDC45*, *MCM5*, and *DONSON* have subsequently been associated with MGS (Burrage et al. 2015; Fenwick et al. 2016; Reynolds et al. 2017; Vetro et al. 2017). Mutations in, respectively, prereplication complex and preinitiation complex components *MCM4* and *GINS1* cause distinct microcephalic dwarfism syndromes with immunodeficiency and/or adrenal failure (Gineau et al. 2012; Cottineau et al. 2017). Finally, mutations in genes encoding subunits of the replicative DNA polymerases *POLA1*, *POLD1*, *POLD2*, *POLE*, and *POLE2* have implicated components of the active replisome in primordial dwarfism, often with immune deficiency, and in the case of *POLE,* adrenal failure (Pachlopnik Schmid et al. 2012; Thiffault et al. 2015; Frugoni et al. 2016; Logan et al. 2018; Conde et al. 2019; Van Esch et al. 2019).

Each of these genes has been shown to be essential for cellular survival in large-scale functional screens (Supplemental Table S1; Chen et al. 2017) while constraint metrics from the gnomAD consortium suggest that loss of a single copy is unlikely to result in developmental disease (Supplemental Table S2; Karczewski et al. 2020). Recessive pathogenic variants are therefore most likely to lead to perturbation of function more severe than haploinsufficiency but falling short of biallelic loss of function. Consistent with this, previously published variants associated with recessive MPD have included hypomorphic missense or splice altering variants that result in at least one allele with residual function. The recent identification of an intronic splice altering mutation in *POLE* in 12 families exemplifies this, where each affected individual inherited different loss-of-function alleles in *trans* with the same intronic variant (c.1686+32C>G) causing a hypomorphic splicing defect. Hypomorphic alleles such as this may not be annotated as deleterious by variant classifiers, so we considered whether additional genes for MPD could be detected by using a variant classification agnostic approach to identify as yet undiscovered cryptic mutations in essential genes. Here, we report the identification of *PRIM1* as an MPD gene, and demonstrate that mutations reduce cellular PRIM1 protein levels, impairing DNA replication.

## Results

### Identification of biallelic variants in PRIM1

**Figure 1.**
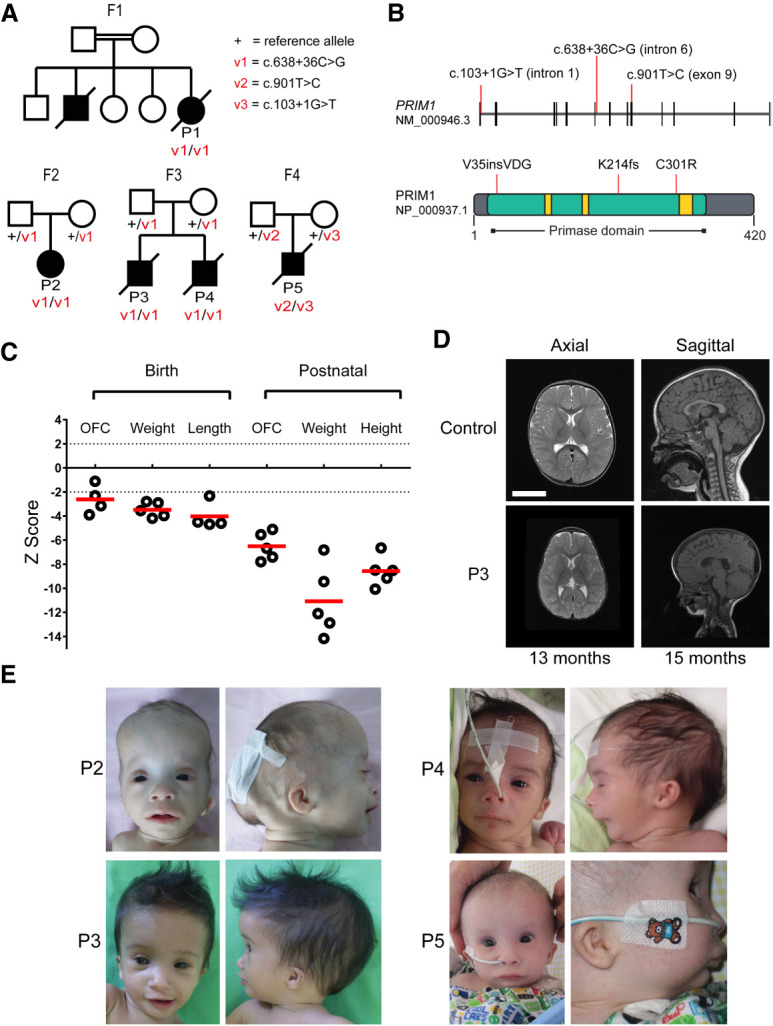
Individuals with biallelic *PRIM1* variants have primordial dwarfism. (*A*) Family pedigrees with segregation of *PRIM1* variants as indicated. (Square) male; (circle) female; (filled symbols) individuals with primordial dwarfism, (strike through) deceased. (*B*) Schematic of *PRIM1* transcript and protein. (Vertical black lines) exons. Locations of variants are indicated by red lines. (Green) Primase domain (NCBI CDD cd04860), (yellow) nucleotide-binding residues. (*C*) Growth parameters of individuals with PRIM1 deficiency. *Z* scores (standard deviations from population mean for age and sex). Dashed lines indicate 95% confidence interval for general population. (Circles) individual subject data points, (red bars) mean values. (*D*) MRI neuroimaging of P3 demonstrates microcephaly with simplified gyri. Axial T2; Sagittal T1. Comparison with age-matched healthy controls. Scale bar, 5 cm. (*E*) Photographs of individuals P2–P5. (P2) 8 mo, (P3) 7 mo, (P4) newborn, (P5) 7 mo. Written consent was obtained from families for photography.

We analyzed whole-genome (WGS) and whole-exome (WES) sequence data from 220 families with microcephalic dwarfism spectrum disorders (occipital–frontal circumference [OFC] ≤−4 SD; height ≤−2 SD) to find variants enriched in this cohort relative to the general population and inherited in a pattern consistent with recessive inheritance (Materials and Methods; Supplemental Table S3). This identified three families sharing a homozygous intronic variant (c.638+36C>G) (Fig. 1A,B) in *PRIM1*, which encodes the catalytic subunit of DNA primase.

Supporting its pathogenicity, the intronic PRIM1 variant was significantly enriched in our microcephalic dwarfism cohort in comparison with the Genome Aggregation Database where just two heterozygotes out of 141,456 individuals were observed (gnomAD v2.1.1, *P* = 3.61 × 10^−15^, all populations, combined WES and WGS data) (Supplemental Table S4).

Inbreeding coefficients in all three families were consistent with parental relatedness (Supplemental Table S5) while kinship estimates confirmed the different families were not closely related (Supplemental Table S6). As well, P1–P4 had large overlapping regions of homozygosity between 10.4 Mb and 32.7 Mb in length on chromosome 12 containing the *PRIM1* gene (Supplemental Fig. S1). These findings provided additional genetic support for *PRIM1* being the causative gene as they were consistent with the expectation for consanguineous families with a rare recessive disease that a disease-causing variant is expected to lie within a region of homozygosity in each family (Lander and Botstein 1987). Within the shared region of homozygosity there was also a common 2-Mb haplotype surrounding the PRIM1 variant in all four affected individuals (Supplemental Fig. S1; Supplemental Table S7). This indicates that all three families share a distant common ancestor (Supplemental Fig. S1; Supplemental Table S5), establishing the causal mutation to be within the haplotype and excluding the only other enriched variant in families 2 and 3, a missense change in *OR6C4*.

Analysis of the c.638+36C>G variant with SpliceAI (Jaganathan et al. 2019), NNSplice (Reese et al. 1997), and MaxEntScan (Yeo and Burge 2004) predicted activation of a cryptic donor site, resulting in the inclusion of 31 nucleotides of intronic sequence following exon 6, expected to disrupt the open reading frame. This, alongside PRIM1 function in DNA replication, provided further support for disruption of PRIM1 causing MPD.

To identify additional disease-causing *PRIM1* alleles, we searched exome data from a subset of 149 trios and singletons from the Deciphering Developmental Disorders (DDD) Project selected on the basis of morphometric criteria (OFC ≤−3 SD and height ≤−3 SD) and absence of previous diagnostic variants identified by DDD. This identified a single individual (P5) compound heterozygous for an essential splice donor variant (c.103 + 1G > T) and a missense variant (c.901T>C, p.Cys301Arg, hereafter referred to as C301R). These variants were extremely rare or absent from public databases, respectively (Table 1). Additional analysis of exome data from P5 did not reveal any other likely diagnostic variants as an alternative explanation. The c.103+1G>T variant results in loss of the donor site of intron 1 while the c.901T>C variant alters a cysteine residue, conserved in vertebrates, to a physiochemically dissimilar arginine. This substitution was predicted damaging by in silico tools including PolyPhen2, SIFT, MutationTaster and CADD (CADDv1.6 Phred score = 28.4).

**Table 1.**
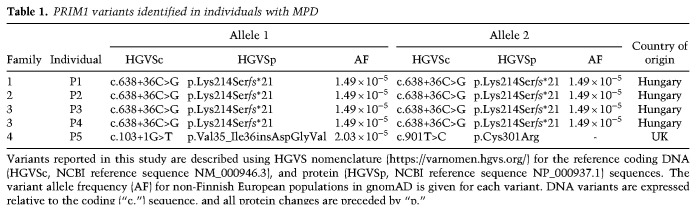
PRIM1 variants identified in individuals with MPD

### PRIM1 subjects share a distinct clinical phenotype

A similar clinical phenotype was evident in all five individuals. All had extreme growth failure, with in utero growth retardation (birth weight −3.4 ± 0.6 SD) and severe postnatal growth restriction (height −8.8 ± 2.0 SD) (Fig. 1C; Supplemental Table S8). While relatively macrocephalic, in absolute terms there was extreme microcephaly (OFC −6.0 ± 1.5 SD). On neuroimaging this was reflected by a “microcephaly with simplified gyri” appearance (Fig. 1D). A common facial appearance was apparent, with prominent forehead and triangular face, with blepharophimosis ± microphthalmia, micrognathia, and small low-set ears (Fig. 1E). Absence of subcutaneous fat, and distally tapered fingers were noted in all affected individuals on clinical examination. Hypothyroidism was frequent alongside significant haematological/immune dysfunction. All had hypo/agammaglobulinemia (Table 2). In four of five cases there was persistent lymphopenia accompanied by intermittent anemia/thrombocytopenia. Documented episodes of sepsis occurred in several patients. In addition, episodes of fever without an identified infective source were recorded. All individuals had chronic respiratory symptoms, and four died in early childhood from either respiratory or GI infections. Hepatic dysfunction was noted clinically, and at postmortem, hepatic fibrosis, cirrhosis, or macronodular regeneration was evident in three of these individuals, suggesting preceding chronic liver inflammation.

**Table 2.**
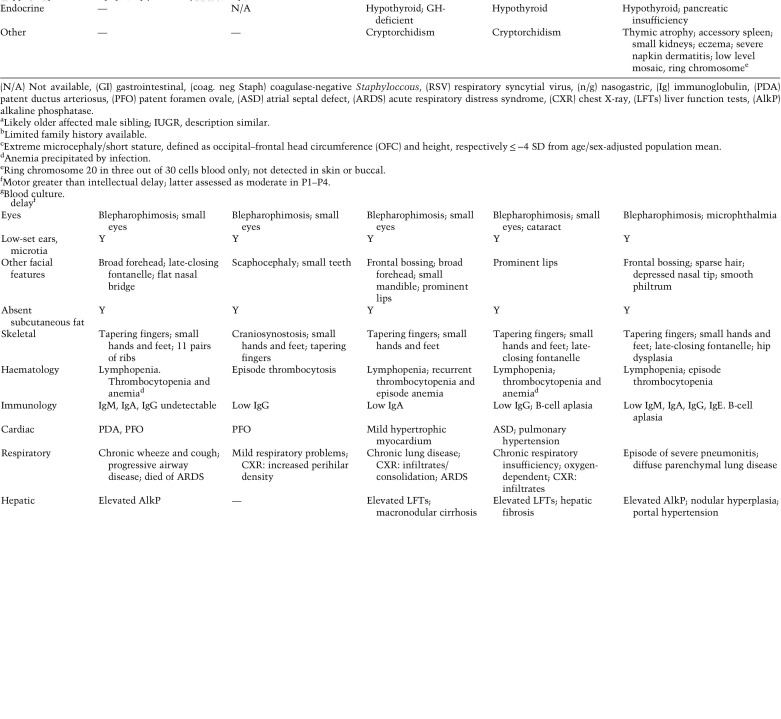
Phenotype and clinical features of PRIM1 subjects

The distinct clinical phenotype in these individuals was consistent with a shared genetic etiology, and taken together with our genetic data, led us to conclude that biallelic mutations in the *PRIM1* gene cause MPD. Next, we sought to test the functional impact of the variants identified in *PRIM1*.

### The c.638+36C>G variant activates a cryptic splice site reducing cellular PRIM1 protein

**Figure 2.**
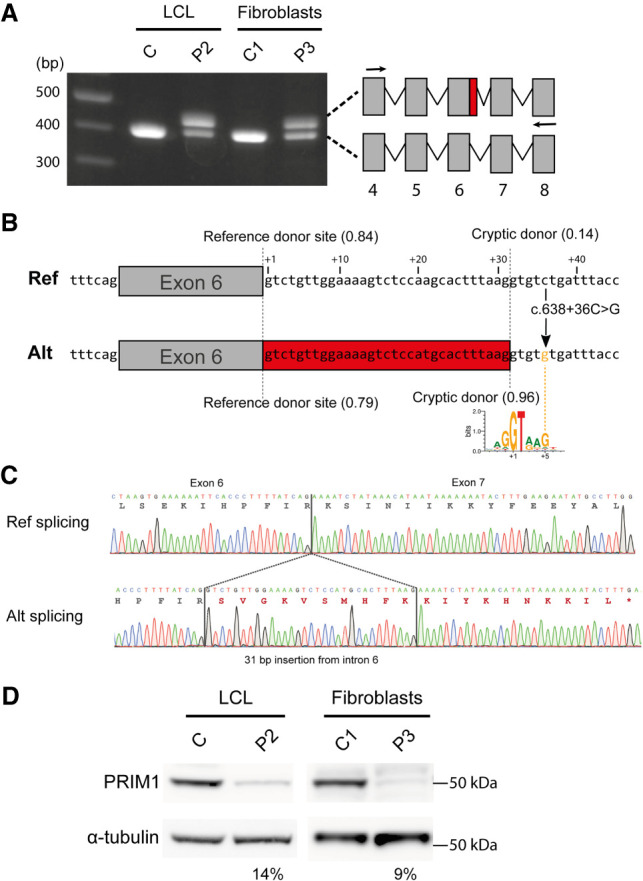
The c.638+36C>G variant creates a cryptic splice donor site, resulting in markedly reduced cellular PRIM1 protein levels. (*A*) The c.638+36C>G variant alters *PRIM1* splicing by creating a cryptic splice donor site in intron 6. RT-PCR of *PRIM1* transcripts from lymphoblastoid cells from P2 and primary fibroblasts from P3. Arrows in schematic indicate the position of primers. (Gray boxes) exons, (red box) retained sequence resulting from missplicing. (*B*) Schematic depicting the effect of the c.638+36C>G variant on splicing of intron 6 of *PRIM1*. The reference and alternate sequences of intron 6 shown with positions of reference and cryptic splice donor sites marked by dotted lines. SpliceAI scores for the donor sites are in brackets. Thirty-one nucleotides included as a result of the c.638 + 36C > G variant are shown on a red background. A sequence logo created with 100,000 randomly selected human U2 splice donor sites from Ensembl (v83) (Cunningham et al. 2015) using WebLogo (Crooks et al. 2004) illustrates how the c.638+36C>G substitution creates a strong splice consensus sequence by providing a G at the +5 position. (*C*) Representative Sanger sequencing traces of splice products relating, respectively, to the lower band in *A* (“ref splicing”) and the alternatively spliced upper band (“alt splicing”). (*D*) PRIM1 protein levels are markedly reduced in cells from individuals P2 and P3, homozygous for the c.638+36C>G variant. Immunoblots of total cell extracts from lymphoblastoid cells (P2) and primary fibroblasts (P3). α-tubulin, loading control. (C) lymphoblastoid, (C1) fibroblast cell lines from control subjects. Quantification of PRIM1 protein levels for P2 and P3 cells relative to C and C1 controls, respectively (normalized to α-tubulin loading control), is shown *below* each blot.

To confirm that the c.638+36C>G variant resulted in missplicing, RT-PCR was performed on RNA extracted from a lymphoblastoid cell line (LCL) established from individual P2 and from primary fibroblasts cultured from individual P3. This demonstrated the presence of an additional PCR product in P2 and P3 corresponding in size to the predicted misspliced transcript (Fig. 2A). Sanger sequencing of these PCR products confirmed in silico splice predictions, with the presence of an additional 31 nt of intronic sequence (Fig. 2B,C), leading to substitution of a lysine to serine at codon 214 and a frameshift with premature termination 21 amino acids later (HGVS nomenclature: p.Lys214Serfs*21, referred to hereafter as K214fs). This would be anticipated to lead to nonsense-mediated decay (NMD) of this transcript, with any remaining translated truncated protein not containing key catalytic residues and therefore enzymatically inactive (Fig. 1B). Reduced cellular levels of full-length protein were therefore expected, and consistent with this, marked reduction in full-length PRIM1 protein levels was evident on immunoblotting of cell extracts from P2 and P3 in comparison with control cell lines (14% of wild-type levels in patient LCL and 9% in fibroblasts) (Fig. 2D). (A band at ∼25 kDa, that might correspond to a nonfunctional truncated PRIM1 protein [27 kDa], was seen inconsistently in blots from P2 LCL extracts but not in P3 fibroblasts [Supplemental Fig. S2].)

### C301R and c.103+1G>T variants also reduce PRIM1 protein levels

P5 was compound heterozygous for c.103+1G>T and C301R variants. However, a patient cell line was not available; therefore, the consequences of these variants were assessed by different methodologies. Our initial expectation was that the essential splice site mutation would be a null allele and the missense variant, partial loss of function.

**Figure 3.**
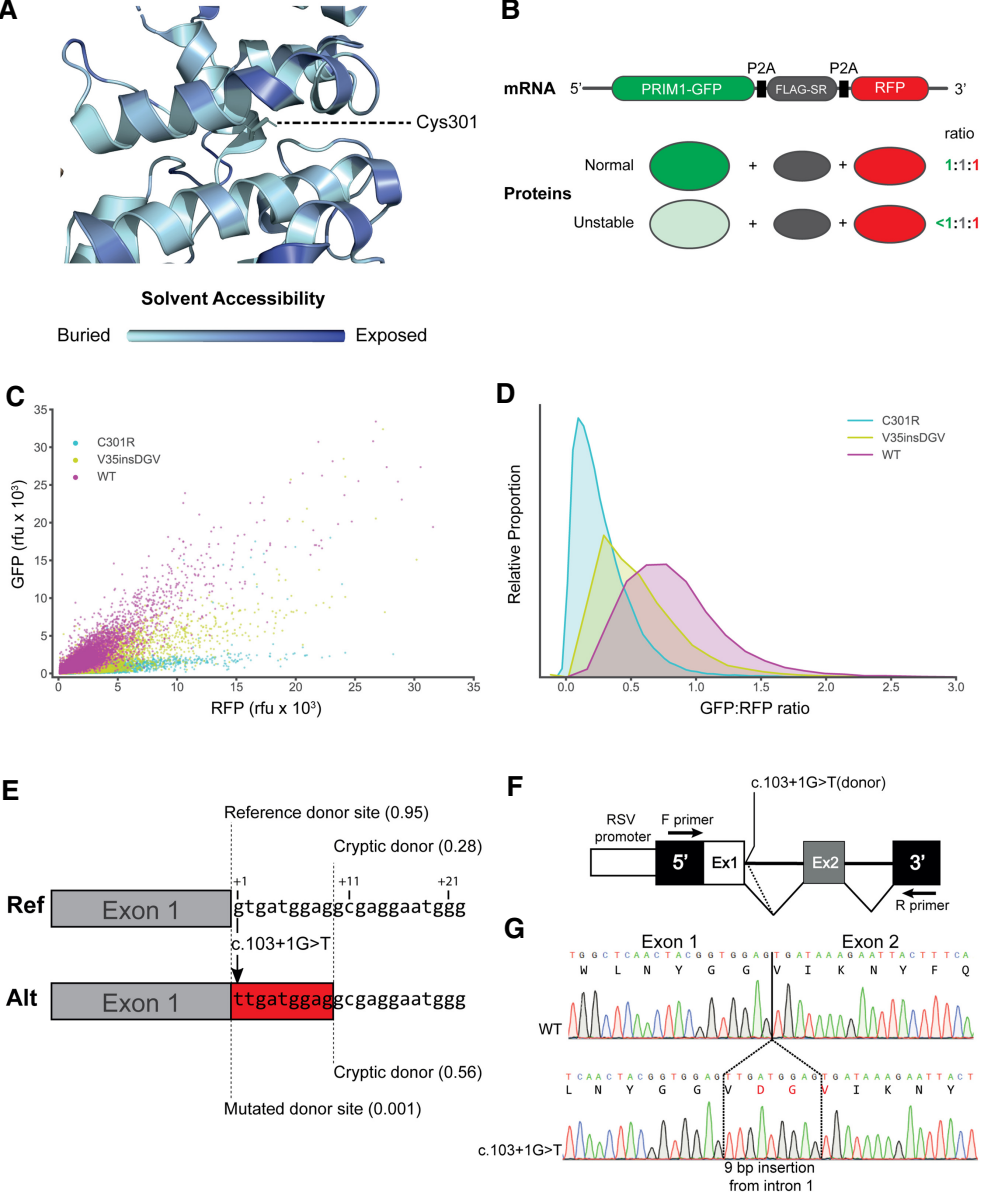
The c.103+1G>T and C301R variants reduce PRIM1 protein levels. (*A*) Cysteine 301, substituted to arginine in P5, lies in a buried hydrophobic region. DNA primase dimer crystal structure (PDB: 4BPU) with residues shaded according to solvent accessibility. (*B*) Schematic of the FACS-based dual-reporter stability assay. Expression vector expresses an mRNA encoding PRIM1-GFP-P2A-FLAG-SR-P2A-RFP. Intervening P2A “self-cleaving” peptide sequences produce PRIM1-EGFP, FLAG-SR, and RFP polypeptides in equimolar amounts. PRIM1-GFP (wild type and mutants) and RFP levels are assayed in individual cells by flow cytometry. PRIM1-GFP and FLAG-SR levels can be independently assessed by immunoblotting (see Supplemental Fig. S3D). (*C*) GFP-RFP scatter plot for wild-type, C301R, and V35insDGV dual reporter constructs. *n* = WT, 27,027; C301R, 44,863; V35insDGV 46,441 cells respectively. (rfu) Relative fluorescence units. (*D*) Kernel density estimation plot of GFP:RFP ratios from *C*. (*E*) Schematic depicting the consequence of the c.103+1G>T variant on splicing. Reference and alternate sequences of intron 1 are shown with positions of the reference and cryptic splice donor sites marked by dotted lines. SpliceAI scores for splice donor sites in brackets. (Red box) Nine nucleotides of intron 6 included as a result of activation of the cryptic splice donor variant. (*F*) Schematic assaying the effect of the c.103+1G>T variant. Minigene assay. DNA spanning exon 1 (Ex1) and exon 2 (Ex2) of *PRIM1* was cloned into the minigene. (Arrows) Position of PCR primers, (dotted line) splicing from cryptic splice donor. (*G*) Representative cDNA Sanger sequence traces from wild-type (WT) and c.103+1G>T variant minigene constructs. The three-amino-acid (DGV) insertion from the c.103+1G>T variant marked in red.

C301 is a residue in a buried hydrophobic region of the protein (Fig. 3A), and its substitution with a bulky, positively charged arginine residue (C301R) was predicted to be highly destabilizing by FoldX (Supplemental Fig. S3; Schymkowitz et al. 2005). We therefore assessed the effect of this substitution on PRIM1 protein levels, developing a FACS-based assay to measure the level of PRIM1-GFP containing this insertion relative to a cotranslated RFP control (Fig. 3B). This approach corrects for cell to cell variation in transfection efficiency and ensures that assessment of protein expression is not confounded by transcriptional differences or mRNA stability. This assay confirmed substantially reduced PRIM1 protein levels for the C301R substitution (25 ± 0.3%) (Fig. 3C,D).

We also modeled this mutation in budding yeast, where substitution of the equivalent residue in *S. cerevisiae* Pri1p to arginine (L309R) also led to a reduction in protein level (Supplemental Fig. S4), whereas the wild-type human cysteine residue (L309C) had no effect on either the level of Pri1 protein (Supplemental Fig. S4) or growth (Supplemental Fig. S5). Similar degradation dynamics of wild-type and L309R Pri1p in cycloheximide chase experiments (Supplemental Fig. S4) suggest that reduced protein levels are most likely the consequence of incorrect folding of the C301R/L309R protein rather than an increase in turnover. Notably C301 is in close proximity to the key catalytic residue R304 (Kirk and Kuchta 1999), suggesting that it might not solely act to reduce protein levels. Consistent with this, the L309R mutation was lethal in yeast (Supplemental Fig. S5), raising the possibility that C301R in human PRIM1 is also a null allele, as disturbance of this catalytic site could potentially lead to loss of function in any mutant protein that persists in the cell.

The C301R substitution appeared to be of greater functional severity than expected, so we examined the c.103+1G>T variant in more detail, considering whether it might instead be hypomorphic in effect. Consistent with this possibility, a minigene assay established that the c.103+1G>T variant could lead to the use of a nearby cryptic splice site (Fig. 3E; Supplemental Fig. S6) resulting in a three-amino-acid in-frame insertion in the PRIM1 transcript (“V35insDGV”) (Fig. 3F,G). The FACS-based stability assay established that the V35insDGV insertion also lowers PRIM1 protein levels (52 ± 0.5% of wild-type levels) (Fig. 3C,D), but less severely than C301R, consistent with this being a partial loss of function mutation.

In conclusion, these experiments confirmed that both variants have functional impact on the PRIM1 protein, that in combination would still leave residual PRIM1 activity.

### Fork stability and origin firing is impaired in PRIM1 primary fibroblasts

Human *PRIM1* encodes the 49 kDa catalytic subunit of the DNA primase heterodimer, which forms a heterotetrameric complex with the two DNA polymerase α subunits (Czechowska and Błasiak 2005). PRIM1 is responsible for the synthesis of short RNA primers required for the initiation of DNA replication and Okazaki fragment synthesis, before handover of these primers to Pol α for DNA synthesis (Rowen and Kornberg 1978; Frick and Richardson 2001). As such, depletion of PRIM1 would be expected to impact the efficiency of DNA replication.

**Figure 4.**
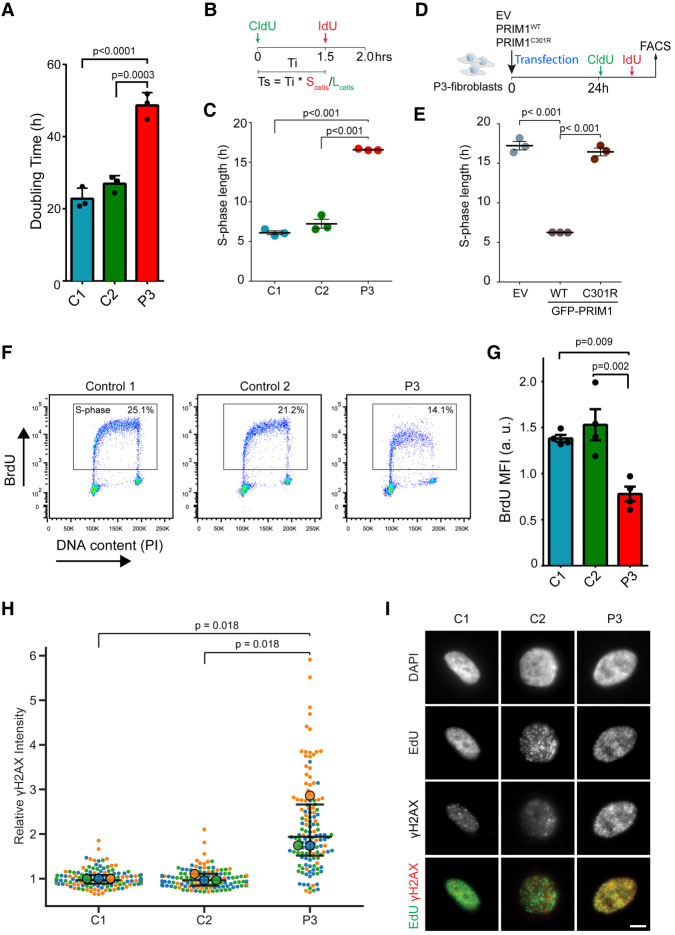
Reduced cell proliferation and impaired DNA replication in PRIM1-deficient primary fibroblasts. (*A*) Cell doubling time plotted for three independent experiments on P3 and two unrelated control C1 and C2 primary fibroblast cell lines. Bars indicate the mean. Error bars indicate SD. (*B*) Schematic of CldU/IdU double-pulse experiment used to determine S-phase time. Cells were labeled with CldU at t = 0, followed by IdU after 1.5 h. Cells leaving S phase (L_cells_) are labeled with CldU only, while cells remaining in S phase (S_cells_) are labeled with both CldU and IdU. (Ts) S-phase length, the product of interval between pulses (Ti) and the proportion of S_cells_ to L_cells_ (Martynoga et al. 2005). (*C*) S-phase time is substantially increased in P3 fibroblasts compared with controls. Mean ± SEM, *N* = 3 experiments. (*D*) Schematic of rescue experiment. P3 fibroblast transfected with either empty vector (EV), wild-type (WT) or C301R-PRIM1-GFP as indicated and after 24 h S-phase time determined as in *B* for GFP + ve cells. (*E*) Complementation with WT PRIM1-GFP rescues slow S-phase progression in P3 fibroblasts. S-phase length plotted for *n* = 3 experiments. Mean ± SEM. (*F*) DNA content and BrdU flow cytometry scatter plots, representative of four independent experiments on control (C1 and C2) and P3 primary fibroblast cell lines. (*G*) BrdU incorporation is reduced in PRIM1-deficient cells during S-phase. Quantification of BrdU mean fluorescence intensity (MFI) from control and patient-derived fibroblasts according to S-phase gate in *F*. (a.u.) Arbitrary units. (*H*) γ-H2AX is increased in S-phase P3 fibroblasts. Mean γ-H2AX intensity calculated for EdU-positive nuclei from C1, C2, and P3 cells. *n* = 3 experiments. Data points are colored by experiment. (Filled circles) Mean values for each replicate, (bars) median and interquartile range (all values). Values were normalized for each experiment relative to C1 mean value. (*P*-values) Repeat measures ANOVA with Tukey multiple comparison test. (*I*) Representative immunofluorescence images of S-phase nuclei quantified in *H*. Scale bar, 5 µm. Statistics in *A*, *C*, *E*, and *G* are one-way ANOVA with Tukey multiple comparison test.

Doubling times of P3 primary fibroblasts were significantly increased compared with controls (P3 = 49 ± 3.6 h, C1 = 23 ± 2.9 h, C2 = 27 ± 2.3 h, *P* < 0.001) (Fig. 4A), and S-phase length, measured by sequential CldU/IdU pulse labeling (Martynoga et al. 2005), was substantially increased (P3 = 16.6 ± 0.12 h, C1 = 6.1 ± 0.4 h, C2 = 7.2 ± 1 h) (Fig. 4B,C). Complementation by transient transfection of wild-type, but not C301R mutant PRIM1 confirmed that the lengthened S-phase was specifically due to PRIM1 deficiency in patient cells (Fig. 4D,E). Impaired efficiency of DNA replication was also observed in BrdU-DNA content flow cytometry, which demonstrated reduced BrdU incorporation in P3 primary fibroblasts during S phase compared with control fibroblast lines (*P* < 0.001) (Fig. 4F,G). In addition, γH2AX levels, a marker of DNA damage, were significantly increased in patient fibroblasts during S phase (Fig. 4H), as were levels of chromatin bound RPA (Supplemental Fig. S7), consistent with the presence of endogenous replication stress. However, whether S-phase checkpoint activation occurs remains to be determined.

FACS analysis of the cell cycle also demonstrated an increased proportion of G1-phase cells (Fig. 4F; Supplemental Fig. S8), without significant differences in the fraction of cleaved caspase-3- or p21-positive patient cells (Supplemental Fig. S9). We therefore concluded that the hypoproliferative phenotype was most likely the consequence of delayed cell cycle progression rather than increased apoptosis or cell cycle exit.

**Figure 5.**
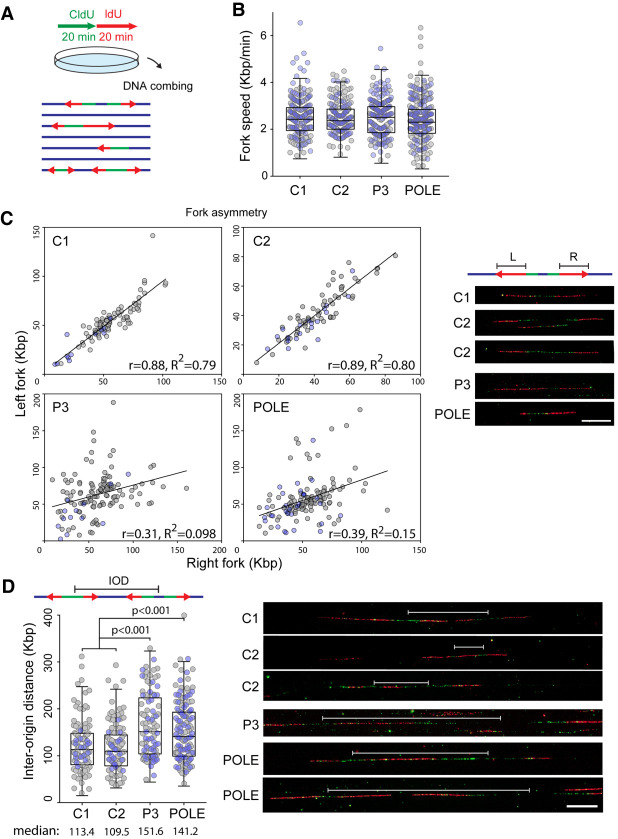
PRIM1 deficiency causes fork asymmetry and increased interorigin distances, consistent with replication fork stalling and decreased replication initiation. (*A*) Schematic depicting DNA combing experiments. Sequential 20 min CldU and IdU pulses were performed on cultured primary fibroblasts, which were then harvested, with DNA combing performed to characterize DNA replication at the single-molecule level. (*B*) DNA fork speed, kilobases per minute, in primary fibroblasts from two unrelated control individuals (C1 and C2), a PRIM1-deficient individual (P3), and a POLE-deficient individual. (*B*–*D*) Gray and blue dots represent measurements from *n* = 2 respective independent experiments. (*C*) PRIM1-deficient cells exhibit fork asymmetry, similar to POLE-deficient cells. Left (L) versus right (R) fork ratio scatter plots. (r) Pearson correlation coefficient. (*Right*) Representative images of bidirectional forks. Scale bar, 20 µm. (*D*) Interorigin distance (IOD) in control, PRIM1-deficient, and POLE-deficient primary fibroblasts; box plots were graphed according to Tukey method (B,D). *P*-values, Kruskal–Wallis test. (*Right*) Representative images of IODs. Scale bar, 20 µm.

We used DNA fiber combing to further characterize replication defects in PRIM1-deficient patient cells. Primary fibroblasts from P3 demonstrated similar replication defects to fibroblasts from an individual with POLE-IMAGe syndrome caused by Pol ε deficiency (P1 from Logan et al. 2018). Fork speed was not affected in either PRIM1- or POLE-deficient cell lines (Fig. 5B), but both cell lines displayed a similar, high degree of perturbed fork-progression compared with controls (Fig. 5C), indicative of elevated fork stalling and restart events, as might be expected for an enzyme continually required for priming of Okazaki fragments during lagging strand synthesis. Increased interorigin distances were also observed (median IOD 152 kb vs. 113 kb, *P* < 0.001) (Fig. 5D), suggesting reduced DNA replication initiation at origins. These alterations in replication dynamics were similarly observed in P2 LCLs (Supplemental Fig. S10).

Taken together, these findings were consistent with impaired replication fork formation and increased replication fork stalling in PRIM1-deficient cells.

## Discussion

We report here the identification of five individuals with biallelic deleterious variants in *PRIM1* using a variant classification independent approach. PRIM1 is essential to cellular survival (Chen et al. 2017) and, like other replisome components, knockout in mice is lethal during embryogenesis (Blake et al. 2011). Mutations in essential genes that result in developmental disorders are necessarily hypomorphic alleles. Identification of such variants is challenging, particularly as they may often be in noncoding regions (Cottineau et al. 2017; Logan et al. 2018; Tarnauskaitė et al. 2019). Consequently, they may not appear on variant lists filtered by functional consequence. Therefore, the agnostic approach used in this study may have utility for the identification of further genes for other Mendelian diseases.

PRIM1 deficiency results in similar growth restriction to other replication-associated disorders such as those associated with Pol α and Pol ε deficiency (Supplemental Figs. S11, S12). Microtia and lymphopenia are also reminiscent of other replisome disorders (Logan et al. 2018; Conde et al. 2019). Likewise, reduced subcutaneous fat in PRIM1-deficient individuals parallels the lipodystrophy found in *POLD1* cases (who had normal growth) due to heterozygous single-codon deletion of the catalytic site of Pol δ (Weedon et al. 2013). However, there are distinctive clinical features for individuals with PRIM1 deficiency, particularly early childhood mortality occurring in four out of five cases. While this might be accounted for by the significant B-cell lymphopenia and accompanying hypo/agammaglobinemia, other disease processes may have also contributed. Chronic lung disease, recurrent diarrhea, and liver dysfunction could also reflect a persisting inflammatory state. Notably a specific intronic variant in *POLA1* causing an X-linked reticulate pigmentary disorder, is also associated with recurrent pneumonias and chronic diarrhea, with underlying immunodeficiency alongside autoinflammation (Starokadomskyy et al. 2016). However, the *POLA1* associated reticulate pigmentary skin phenotype is not reported in any of the individuals with *PRIM1* variants identified herein. Most individuals with MPD caused by *POLA1* variants have growth retardation and microcephaly without such features (Van Esch et al. 2019), and facially *PRIM1* deficiency appears distinct from these and individuals with *POLE*-associated IMAGe syndrome (Logan et al. 2018). Likewise, blepharophimosis, microphthalmia, hypothyroidism, episodic thrombocytopenia, and anemia do not appear to be features reported in other replisome disorders.

In conclusion, we describe a distinct form of microcephalic dwarfism associated with *PRIM1* mutations. Similar to POLE/A1 DNA polymerase disorders, fork defects are seen in PRIM1-deficient cells (Bellelli et al. 2018; Van Esch et al. 2019). This likely reflects an inability of these cells to establish sufficient replication origins and sustain lagging strand synthesis at a sufficient rate to meet the demand for rapid replication. The ensuing delay to the cell cycle and reduced cellular proliferation provides a likely mechanism for the resulting “hypocellular” microcephalic dwarfism. Given the necessity of several polymerases to act in concert in every replicating cell, the different phenotypic manifestations of Pol α-, Pol δ-, Pol ε-, and DNA primase-related disorders are surprising and require future investigation.

## Materials and methods

### Research subjects

Genomic DNA was extracted from blood samples by standard methods or from saliva samples using Oragene collection kits according to the manufacturer's instructions. Informed consent was obtained from all participating families. This study was approved by the Scottish Multicentre Research Ethics Committee (REC; 05/MRE00/74). Parents provided written consent for the publication of photographs of the affected individuals.

The DDD study has UK Research Ethics Committee approval (10/H0305/83, granted by the Cambridge South REC, and GEN/284/12 granted by the Republic of Ireland REC).

### Whole-genome sequencing and variant calling

Whole-genome sequencing for P1, P2, and P4 was performed by Edinburgh Genomics (Clinical Division) as part of the Scottish Genomes Project (SGP) to 30× coverage using TruSeq nano library preparation kits and a HiSeq X sequencing platform (Illumina). FastQs generated by Edinburgh Genomics were aligned to the human genome (hg38, including alt, decoy, and HLA sequences) using bwa mem (0.7.13) (Li 2013). Postprocessing was performed with samblaster (0.1.22) (Faust and Hall 2014) to mark duplicate reads, and the genome analysis toolkit (GATK, v3.4-0-g7e26428) (McKenna et al. 2010) for indel realignment and base recalibration. Genotype likelihoods for each sample were calculated using the GATK HaplotypeCaller and resulting GVCF files were called jointly using GATK's GenotypeGVCFs function. Variant quality score recalibration (VQSR) was performed as per GATK best-practices (Van der Auwera et al. 2013) and a truth sensitivity threshold of 99.9% was applied. Functional annotations were added using Ensembl's Variant Effect Predictor (v90) (McLaren et al. 2016).

### Variant filtering and identification of a variant activating a cryptic splice donor in *PRIM1*

Cohort WGS variant data were filtered to only include variants with an allele frequency < 0.5% in all gnomAD populations at biallelic sites outside of repeat masked or low-complexity regions using VASE (v0.1, https://github.com/david-a-parry/vase). Remaining variants were analyzed against gnomAD v2.1 data converted to hg38 coordinates.

Where variants were present in gnomAD WES and WGS VCFs, allele counts per population were extracted from VCFs. For variants absent from either or both gnomAD WES or WGS VCFs, homozygous reference allele counts were derived for the respective samples using sequencing depth data, where the fraction of individuals with sequencing depth of ≥5 were counted as homozygous reference. Variants were annotated with *P*-values from one-tailed Fisher's exact tests comparing the alternate/reference allele counts in nonrelated affected individuals in the MPD cohort versus individual population groupings in gnomAD.

Annotated variants were assessed for segregation consistent with recessive inheritance. At least one allele was required to have *P*-values <5 × 10^−5^ for each of the African/African American, Latino/Admixed American, East Asian, Finnish, non-Finnish European, and South Asian gnomAD subpopulations. The second allele for a gene was required to either match these same criteria or to be a predicted loss-of-function variant. Analysis was restricted to canonical transcripts and biallelic combinations of alleles observed in nonaffected family members were excluded. Where available, phase information from parental genotypes and/or physical phasing was used to exclude biallelic combinations in *cis*.

This enrichment strategy could be confounded by differing variant calling sensitivity in gnomAD compared with the MPD cohort and by population stratification. In order to mitigate these effects we required enriched alleles to be below the given *P*-value threshold in each of the population groups listed above and then ranked genes based on incidental occurrences of variants within the MPD cohort, gene essentiality, and the maximum (least significant) observed *P*-value. Incidental occurrences were defined as the number of families where an individual was a carrier for a variant, yet no qualifying second allele was observed in the affected individual(s). Additionally, more than two qualifying alleles in the same family were also defined as incidental occurrences to address the presence of “noisy” genes. Gene essentiality was defined as the fraction of studies in OGEE (Chen et al. 2017) in which a gene was found to be cell-essential. A ranking score for variants was calculated as follows: *s* = (1 + *oi*)(1 + *fi*)(1.1 − *ess*), where “*oi*” indicates the number of incidental occurrences in other families, “*fi*” indicates the number of incidental occurrences within the same family, and “*ess*” represents the fraction of studies in which the gene is considered cell essential. A lower score was considered “better” such that variants were penalized for incidental occurrences and lack of evidence for gene essentiality.

These methods were implemented in a Python package (ReVERSe), available at https://git.ecdf.ed.ac.uk/dparry/reverse.

### Segregation filtering of DDD CAPS14 cohort

VASE (v0.1, https://github.com/david-a-parry/vase) was used to perform variant filtering and segregation analysis. Variants overlapping *PRIM1* were filtered to remove those with a frequency >0.5% in gnomAD or dbSNP151. Additionally, those variants not predicted to be either protein-altering variants, within a splice region, or with a SpliceAI (Jaganathan et al. 2019) δ score ≥0.5 were also excluded. Genotype calls were filtered if samples had fewer than five reads covering the site or the fraction of variant reads to total reads (VAF) were <0.25. Remaining variants were assessed for familial segregation consistent with recessive inheritance.

Following identification of *PRIM1* compound heterozygous variants in F5, variant data for this family were reanalyzed to test exome-wide for any other possible causative variants. Data for this family were analyzed exome-wide for recessive variants as above and also analyzed for de novo variants, requiring a VAF of ≥0.25 in the proband and VAFs of <0.05 in both parental genotypes and total site depths of ≥8 in parents and proband.

### Plasmid construction

All oligonucleotides and plasmids used in this study are listed in Supplemental Tables S9 and S10, respectively. Plasmids pMAR780 and pMAR781 were generated by cloning annealed oligonucleotides into BclI/SwaI-digested pML104 and pML107 plasmids, respectively (Laughery et al. 2015). To construct pMAR782, the *PRI1* (*YIR008C*) coding sequence, without stop codon and with a silent PAM site mutation (c.1945G > C), was amplified by PCR using MRY201 genomic DNA as template and cloned into XbaI/BamHI digested pGFP-C-FUS (Niedenthal et al. 1996). pMAR782 allows expression of C-terminally GFP-tagged Pri1 protein in medium lacking methionine. To construct pMAR790, a 1.35-kb fragment of human genomic DNA (chr12:56750975-56752323, hg38) covering *PRIM1* exon 1 and exon 2 was PCR-amplified and cloned into BspEI/XbaI sites of RHCglo (a gift from Thomas Cooper; Addgene plasmid 80169) (Singh and Cooper 2006), fusing *PRIM1* exon 1 with exon 1 of the minigene construct. The donor (c.103+1G>T) splice site mutation was introduced in intron 1 using site-directed mutagenesis (NEB Q5 site-directed mutagenesis kit), generating pMAR793. Human C-terminally EGFP-tagged PRIM1 was synthesized by IDT flanked by NheI and BspEI sites and provided in pUCIDT-AMP GoldenGate (pMAR796). The NheI/BspEI fragment was then transferred into pmGFP-P2A-K0-P2A-RFP (a gift from Ramanujan Hegde; Addgene plasmid 105686) (Juszkiewicz and Hegde 2017), replacing mGFP and creating pMAR797. To introduce C301R and VDGins mutations, Q5 SDM was performed on pMAR796, creating pMAR798 and pMAR802, respectively, and the sequence-verified PRIM1-EGFP NheI/BspEI fragments cloned into pmGFP-P2A-K0-P2A-RFP to make pMAR799 and pMAR803. All plasmids were confirmed by restriction digest analysis and Sanger sequencing.

### Cells and cell culture

Primary dermal fibroblasts were established from skin punch biopsies and maintained in AmnioMAX medium (Thermo Fisher Scientific 17001074) in 5% CO_2_ and 3% O_2_. Patient lymphoblastoid cell lines (LCLs) were generated in house from peripheral blood samples by EBV transformation using standard methods. LCLs were maintained in RPMI 1640 supplemented with 15% FBS, L-glutamine, and penicillin/streptomycin antibiotics. HeLa (ATCC) cells were maintained in DMEM supplemented with 10% FBS and 5% penicillin/streptomycin in 5% CO_2,_ under normoxic conditions. Genotypes of patient cells lines were validated by Sanger sequencing.

### RT-PCR

Total RNA was extracted from cell lines using the RNeasy minikit (Qiagen) according to the manufacturer's instructions. Following DNase I (Qiagen) treatment cDNA was generated using SuperScript III reverse transcriptase (Thermo Fisher Scientific). RT-PCR was performed on cDNA using primers in exons 4 and 8 (Supplemental Table S9).

### Minigene splicing assay

RPE1 cells were seeded (4 × 10^5^ cells/well) in a six-well plate. After 24 h, cells were transfected with 800 ng of pMAR790 or pMAR791 plasmid in Opti-MEM Reduced Serum Medium with Lipofectamine 2000 according to the manufacturer's instructions. Cells were harvested after 24 h, RNA extracted using the RNeasy Mini Kit (Qiagen), and cDNA generated using SuperScript III reverse transcriptase (Invitrogen). PCR amplification was then performed with RSV5U and RTRHC primers, and wild-type and mutant cDNA amplicons resolved by agarose gel electrophoresis to visualize splicing differences. PCR products were cloned into pGEM-T Easy (Promega), and Sanger sequencing was performed on 20 clones.

### Immunoblotting

Whole-cell extracts were prepared by lysis using 50 mM Tris-HCl (pH 8), 280 mM NaCl, 0.5% NP40, 0.2 mM EGTA, 1 mM DTT, and 10% glycerol, supplemented with Roche Complete protease inhibitor cocktail. Protein samples were resolved on 4%–12% NuPAGE Bis-Tris gels (Thermo Fisher Scientific) and transferred to nitrocellulose or PVDF membranes. Subsequent immunoblotting was performed using the following antibodies: anti-p48 Primase (8G10; 1:1000; Cell Signaling 4725), anti-Vinculin (hVIN-1; 1:2000; Sigma-Aldrich, V9264), anti-α-Tubulin (B-5-1-2; 1:10,000; Sigma-Aldrich, T6074), anti-GFP (Living Colors JL-8; 1:4000; Clontech), anti-FLAG (M2; 1:2000; Sigma-Aldrich F1804), or anti-Actin (1:4000; Sigma-Aldrich A2066).

Finally, detection was performed using Amersham ECL Prime Western blotting detection reagent on the ImageQuantLAS4000 (GE Healthcare Life Sciences), or the Odyssey CLx imaging system (LI-COR Biosciences). Quantifications were performed using ImageQuant TL 7.0 and Image Studio Lite 5.2, respectively.

### FACS-based dual-reporter stability assay

C-terminally EGFP-tagged PRIM1 (wild type or mutants) was cloned into a mammalian expression vector as described under Plasmid Construction. Expression from the CMV promoter of the resulting constructs (pMAR797 and derivatives) produces mRNA encoding PRIM1-EGFP-P2A-FLAG-SR-P2A-RFP, which when translated due to intervening P2A “self-cleaving” peptide sequences produces three individual polypeptides in equimolar amounts: PRIM1-EGFP, FLAG-SR, and RFP. This allows comparison of the relative level of PRIM1-EGFP (wild-type and mutants) and RFP in individual cells.

RPE1 cells were seeded (4 × 10^5^ cells/well) in a six-well plate, and a day later transfected with 2 µg of the reporter plasmid in Opti-MEM reduced serum medium with Lipofectamine 2000 per the manufacturer's instructions. At 24 h after transfection, live cells were assayed for the presence of GFP and RFP using a BD Biosciences LSR Fortessa flow cytometer and data were analyzed using FlowJo software (v7.6.1, Tree Star).

### BrdU-DNA content flow cytometry

Primary fibroblasts were seeded into AmnioMAX medium (Life Technologies) to achieve ∼60% confluency after 24 h. Cells were then incubation with 10 μM BrdU for 30 min, washed, harvested, and fixed with 70% EtOH for 16 h at −20°C. Fixed cells were then digested with 1 mg/mL pepsin, denatured in 2 M HCl for 15 min, and washed with PBS. After blocking in 0.5% BSA and 0.5% Tween-20, BrdU labeling was detected using anti-BrdU antibody (1:75; Abcam ab6326) and anti-rat Alexa fluor 488 secondary antibody (Thermo Fisher Scientific A11006). DNA content was determined by costaining with 50 μg/mL propidium iodide. Cells were assayed on a BD Biosciences LSR Fortessa flow cytometer and data were analyzed using FlowJo software (v7.6.1, Tree Star).

### Cell proliferation doubling times

Primary fibroblasts (1.5 × 10^5^ cells) were seeded on day 0 into a T25 flask. Cell counts were performed every 3 d using a MOXI Z Mini automated cell counter (MXZ001). After counting, 1.5 × 10^5^ cells were reseeded into a new T25. Doubling times were calculated during log-phase growth (day 3 to day 12) using the formula *t*/log_2_(*e*/*b*), where *t* = time in hours, *e* = final population size, and *b* = population size at the start of log phase growth.

### S-phase time measurements and PRIM1 complementation (CldU/IdU pulse labeling)

S-phase time (Ts) measurements were performed using sequential pulses of CldU and IdU to determine the fraction of cells that leave S-phase in a specified time window, according to the formula Ts = Ti*S_cells_/L_cells_, where Ti represents the time (hours) between pulses, S_cells_ are the number of cells that are in S phase at the second pulse (all double-positive), and L_cells_ are the number of cells that have left S-phase at the time of the second pulse (CldU-positive and IdU-negative cells).

Primary fibroblasts, cultured on coverslips, were pulsed with 25 µM CldU for 1.5 h, followed by a 30-min 125 µM IU pulse. Cells were then fixed in 4% PFA.

For complementation experiments, P3 fibroblasts were electroporated with dual expression vectors (pmGFP-P2A-K0-P2A-RFP, pMAR797, or pMAR799, expressing GFP, WT-PRIM1-GFP-WT, or C301R-PRIM1-GFP, respectively) using the Invitrogen Neon kit (MPK10096) according to the manufacturer's instructions (2.5 µg of plasmid DNA for 1 × 10^6^ cells, 20-msec pulse width). Twenty-four hours after electroporation, cells were pulsed with CldU and IdU (as above), trypsinized, and sorted on a BD FACSAria to select GFP-positive, successfully transfected cells. Cells were collected in AmnioMAX C-100, seeded on coverslips in 24-well plates for 1.5 h, and subsequently fixed in 4% PFA. Immunofluorescence for CldU and IdU, as outlined below, was performed using rat anti-BrdU (Abcam ab6326) and mouse anti-BrdU (BD 347580).

### Immunofluorescence

Fibroblasts were grown on coverslips for 24 h, fixed with 4% PFA, washed, and blocked for 1 h in 10% goat serum/PBS/0.1% Triton. Independent coverslips were incubated with p21 (1:200; Calbiochem OP64), cleaved caspase 3 (1:1000; Cell Signaling 9661), γH2AX (1:1000; Millipore 05-636), or RPA2 (1:200; Calbiochem NA19L) antibodies. After overnight incubation in primary antibodies, signals were detected using fluorescently conjugated antibodies and costained with DAPI. For all antibodies except cleaved caspase 3 and p21, cells were pre-extracted for 5 min on ice with ice-cold buffer (25 mM HEPES at pH 7.4, 50 mM NaCl, 1 mM EDTA, 3 mM MgCl_2_, 300 mM sucrose, 0.5% Triton X-100) before fixation to remove soluble proteins and detection of chromatin-bound fractions. For γ-H2AX staining, cells were pulsed with 20 µM EdU (Sigma 900584) for 1 h before fixation. EdU was detected subsequent to immunofluorescence using the click reaction and azide-Alexa fluor 488 (Thermo Fisher A10266).

### DNA combing

Primary fibroblast cells exponentially growing in AmnioMAX C-100 (Thermo Fisher Scientific 17001074) medium under hypoxic conditions were pulsed with 25 μM CldU (Sigma C6891) for 20 min, washed with prewarmed PBS, and then pulsed with 125 μM IdU (Sigma I7125) for 20 min. After trypsinization, 5 × 10^5^ cells were used to cast three to four agarose (Bio-Rad 1613111) plugs per condition and processed for DNA combing according to a previously described protocol (Gallo et al. 2016), omitting the SCE buffer plug digestion steps. IdU and CldU were detected using mouse anti-BrdU (BD 347580) and rat anti-BrdU (Abcam ab6326), respectively. DNA was detected using anti-ssDNA antibody (Millipore MAB 3034). Images were acquired on a wide-field microscope (Zeiss Axiophot) with a 63× or 40× lens. The 2.33-kb/µm elongation rate (micron to kilobase conversion) was obtained from bacteriophage λ DNA combing and measurement, as described previously (Gallo et al. 2016). Measurements and analysis were performed using ImageJ. DNA fork speed was obtained dividing the length of the IdU track-adjacent CldU tracks (ongoing forks) by the IdU incubation time (20 min) and is expressed in kilobases per minute. Fork asymmetry is presented as left IdU versus right IdU ratios. Interorigin distances (IODs) correspond to the space (in kilobases) between the center points of adjacent bidirectional replication origins.

### Data access

WGS data from families 1–3 are available on request from the relevant Data Access Committee from the European Genome-Phenome Archive (EGA) under accession number EGAS00001004703.

### The Scottish Genomes Partnership

Members of the Scottish Genome Partnership include Timothy J. Aitman,^9^ Andrew V. Biankin,^10^ Susanna L. Cooke,^10^ Wendy Inglis Humphrey,^9^ Sancha Martin,^10^ Lynne Mennie,^11^ Alison Meynert,^12^ Zosia Miedzybrodzka,^13^ Fiona Murphy,^14^ Craig Nourse,^10^ Javier Santoyo-Lopez,^15^ Colin A. Semple,^12^ Nicola Williams,^16^

## Supplementary Material

Supplemental Material

## Footnotes

9 Centre for Genomic and Experimental Medicine, MRC Institute of Genetics and Molecular Medicine, the University of Edinburgh, Edinburgh EH4 2XU, United Kingdom

10 Wolfson Wohl Cancer Research Centre, Institute of Cancer Sciences, University of Glasgow, Glasgow G61 1QH, United Kingdom

11 University of Aberdeen, Aberdeen AB24 3FX, United Kingdom

12 MRC Human Genetics Unit, MRC Institute of Genetics and Molecular Medicine, the University of Edinburgh, Edinburgh EH4 2XU, United Kingdom

13 Department of Medical Genetics, Ashgrove House, NHS Grampian, Aberdeen AB25 2ZA, United Kingdom

14 National Services Division, NHS National Services Scotland, Edinburgh EH12 9EB, United Kingdom

15 Edinburgh Genomics Clinical Division, the Roslin Institute, University of Edinburgh, Edinburgh EH25 9RG, United Kingdom

16 West of Scotland Genetic Services, Queen Elizabeth University Hospital, Glasgow G51 4TF, United Kingdom
