# Supplementary material for: PRIM1 deficiency causes a distinctive primordial dwarfism syndrome
Source: Genes Dev. 2020 Nov 1;34(21-22):1520–33. doi: 10.1101/gad.340190.120 (PMC7608753; doi:10.1101/gad.340190.120)
Supplement: Supplemental Material [file supp_34_21-22_1520__index.html]

PRIM1 deficiency causes a distinctive primordial dwarfism syndrome — Supplemental Material 

# PRIM1 deficiency causes a distinctive primordial dwarfism syndrome

## Supplemental Material

- Supplemental\_Table\_S3.xlsx
- Supplemental\_Table\_S4.docx
- Supplemental\_Table\_S5.docx
- Supplemental\_Table\_S6.docx
- Supplemental\_Table\_S11.docx
- Supplemental\_Table\_S7.xlsx
- Supplemental\_Table\_S8.docx
- Supplemental\_Table\_S9.docx
- Supplemental\_Table\_S10.docx
- Supplementary\_Text\_and\_Figures.pdf
- Supplemental\_Table\_S1.docx
- Supplemental\_Table\_S2.docx
