# Supplementary material for: PRIM1 deficiency causes a distinctive primordial dwarfism syndrome
Source: Genes Dev. 2020 Nov 1;34(21-22):1520–33. doi: 10.1101/gad.340190.120 (PMC7608753; doi:10.1101/gad.340190.120)
Supplement: Supplemental Material [file supp_gad.340190.120_Supplemental_Table_S4.docx]

| **Population** | **Alternate** | **Reference** | **P-Value** |
| --- | --- | --- | --- |
| MPD Cohort | 6 | 434 | - |
| gnomAD All Populations | 2 | 194,094 | 3.61 x 10^-15^ |
| gnomAD African | 0 | 18,318 | 1.61 x 10^-10^ |
| gnomAD Latino | 0 | 22,966 | 4.27 x 10^-11^ |
| gnomAD East Asian | 0 | 13,432 | 9.85 x 10^-10^ |
| gnomAD European (non-Finnish) | 1 | 82,553 | 1.50 x 10^-13^ |
| gnomAD South Asian | 0 | 22,298 | 5.31 x 10^-11^ |
| gnomAD Ashkenazi Jewish | 0 | 8,076 | 1.84 x 10^-8^ |
| gnomAD Finnish | 0 | 17,242 | 2.30 x 10^-10^ |
| gnomAD Other | 1 | 4,355 | 3.73 x 10^-6^ |

**Supplemental Table S4: Allele counts of the *PRIM1* c.638+36C>G variant in subjects from the MPD cohort and gnomAD population groupings.** P-values for one-sided Fisher’s Exact tests of the MPD cohort versus each gnomAD population grouping are given. This variant was not reported in gnomAD WGS data. Allele counts above were derived from variant calls from the gnomAD WES dataset plus allele counts derived from sequence read depth data for the WGS dataset (see methods).
