# Supplementary material for: PRIM1 deficiency causes a distinctive primordial dwarfism syndrome
Source: Genes Dev. 2020 Nov 1;34(21-22):1520–33. doi: 10.1101/gad.340190.120 (PMC7608753; doi:10.1101/gad.340190.120)
Supplement: Supplemental Material [file supp_gad.340190.120_Supplemental_Table_S5.docx]

| **Family** | **Individual** | **Inbreeding Coefficient** |
| --- | --- | --- |
| F1 | P1 | 0.07231 |
| F2 | P2 | 0.03643 |
| F3 | P4 | 0.08577 |

**Supplemental Table S5: Inbreeding Coefficients.** The inbreeding coefficient (F) was calculated using WGS data from individuals with PRIM1 deficiency.
