# Supplementary material for: PRIM1 deficiency causes a distinctive primordial dwarfism syndrome
Source: Genes Dev. 2020 Nov 1;34(21-22):1520–33. doi: 10.1101/gad.340190.120 (PMC7608753; doi:10.1101/gad.340190.120)
Supplement: Supplemental Material [file supp_gad.340190.120_Supplemental_Table_S6.docx]

| **Individual 1** | **Individual 2** | **Kinship Coefficient (φ)** |
| --- | --- | --- |
| P1 | P2 | -0.02283 |
| P1 | P4 | -0.05057 |
| P2 | P4 | -0.02118 |

**Supplemental Table S6: Relatedness Estimates.** The KING (Manichaikul et al. 2010) algorithm was used to calculate kinship coefficients from WGS data from individuals with PRIM1 deficiency.
