# Supplementary material for: PRIM1 deficiency causes a distinctive primordial dwarfism syndrome
Source: Genes Dev. 2020 Nov 1;34(21-22):1520–33. doi: 10.1101/gad.340190.120 (PMC7608753; doi:10.1101/gad.340190.120)
Supplement: Supplemental Material [file supp_gad.340190.120_Supplemental_Table_S11.docx]

**Supplemental Table S11: Yeast strains used in this study**

| Strain | Genotype | Reference |
| --- | --- | --- |
| BY4741 | *MAT*a *his3Δ1 leu2Δ0 met15Δ ura3Δ0* | (Brachmann et al. 1998) |
| MRY175 | *MAT*a *his3Δ1 leu2Δ0 met15Δ ura3Δ0 PRI1:13myc-HphMX6* | This study |
| MRY201/202 | *MAT*a *his3Δ1 leu2Δ0 met15Δ ura3Δ0 PRI1-WT(V315V; GTG>GTC):13myc-HphMX6* | This study |
| MRY203/204 | *MAT*a *his3Δ1 leu2Δ0 met15Δ ura3Δ0 pri1-L309C:13myc-HphMX6* | This study |
| MRY215 | *MAT*a *his3Δ1 leu2Δ0 ura3Δ0* | This study |
| MRY216 | *MAT*a *his3Δ1 leu2Δ0 ura3Δ0 PRI1:13myc-HphMX6* | This study |
| MRY199 | *MAT*a *his3Δ1 leu2Δ0 ura3Δ0 PRI1-WT(V315V; GTG>GTC)* + pGFP-C-PRI1(V315V) | This study |
| MRY200 | *MAT*a *his3Δ1 leu2Δ0 ura3Δ0* *pri1-L309R* + pGFP-C-PRI1(V315V) | This study |
| MRY205/223/224/225 | *MAT*a *his3Δ1 leu2Δ0 ura3Δ0* *PRI1-WT(V315V; GTG>GTC):13myc-HphMX6* + pGFP-C-PRI1(V315V) | This study |
| MRY206/226/227/228 | *MAT*a *his3Δ1 leu2Δ0 ura3Δ0* *pri1-L309R:13myc-HphMX6* + pGFP-C-PRI1(V315V) | This study |
