# Supplementary material for: PRIM1 deficiency causes a distinctive primordial dwarfism syndrome
Source: Genes Dev. 2020 Nov 1;34(21-22):1520–33. doi: 10.1101/gad.340190.120 (PMC7608753; doi:10.1101/gad.340190.120)
Supplement: Supplemental Material [file supp_gad.340190.120_Supplemental_Table_S8.docx]

| **ID** | **Prenatal Growth** | | | | **Postnatal Growth** | | | |
| --- | --- | --- | --- | --- | --- | --- | --- | --- |
|  | **Gestation /weeks** | **Weight /kg (SD)** | **Length /cm (SD)** | **OFC /cm (SD)** | **Age at exam** | **Weight /kg (SD)** | **Height /cm (SD)** | **OFC /cm (SD)** |
| P1 | 30 | 0.74 (-2.91) | 34 (-2.34*) | 25 (-2.32) | 4.5 months | 1.88 (-6.82) | 40 (-8.49) | 30 (-7.41) |
| P2 | 38 | 1.48 (-3.97) | 40 (-4.68) | 32 (-1.12) | 5 years | 6.095 (-12.88) | 71 (-8.52) | 41 (-7.80) |
| P3 | 35 | 0.99 (-4.19) | 38 (-4.60) | 28 (-3.15) | 9 months | 3 (-9.45) | 55 (-6.65) | 38 (-6.68) |
| P4 | 38 | 1.64 (-3.55) | 40.5 (-4.50) | 29 (-3.90) | 16 months | 2.74 (-14.17) | 54 (-9.16) | 41 (-5.55) |
| P5 | 38 | 1.956 (-2.80) | - | - | 2 years | 3.78 (-12.11) | 55.7 (-10.07) | 43.5 (-5.11) |

**Supplemental Table S8: Anthropometric measurements in individuals with PRIM1 deficiency.** SD, Z-scores calculated using LMS growth, British 1990 dataset. *Z-score calculated using UK WHO preterm dataset as 30-week gestation not available in British 1990 dataset.
