# Supplementary material for: PRIM1 deficiency causes a distinctive primordial dwarfism syndrome
Source: Genes Dev. 2020 Nov 1;34(21-22):1520–33. doi: 10.1101/gad.340190.120 (PMC7608753; doi:10.1101/gad.340190.120)
Supplement: Supplemental Material [file supp_gad.340190.120_Supplemental_Table_S9.docx]

**Supplemental Table S9: Oligonucleotides used in this study**

| Name | Sequence | Description |
| --- | --- | --- |
| PRIM1_c.638+36_F | CACCTTTTAGGAATACTGCAGCA | Forward primer for PCR amplification and Sanger confirmation of the c.638+36C>G variant |
| PRIM1_c.638+36_R | TGCTGCACATGACTGTATTTATATTG | Reverse primer for PCR amplification and Sanger confirmation of the c.638+36C>G variant |
| PRIM1_c.103+1_F | CTGAGCAAGTGTCTGGATGG | Forward primer for PCR amplification and Sanger confirmation of the c103+1G>T variant |
| PRIM1_c.103+1_R | TGGAAATGTGATGCTGCCAG | Reverse primer for PCR amplification and Sanger confirmation of the c103+1G>T variant |
| PRIM1_c.901_1F | TTTCTTAACCCGGCTGCATC | Forward primer for PCR amplification and Sanger confirmation of the c.901T>C variant |
| PRIM1_c.901_1R | AGAACTTCCTGTCAGGGCTG | Reverse primer for PCR amplification and Sanger confirmation of the c.901T>C variant |
| RT PCR primer F | GGCCATACGCATCATTGACAG | Forward primer for amplification of *PRIM1* cDNA (Figure 2A) |
| RT PCR primer R | CCCAACGCTGAAGTGAATTGT | Reverse primer for amplification of *PRIM1* cDNA (Figure 2A) |
| Prim 1 Ex 4 Seq 1F | TGCTGGACCCTCATGACAAT | Internal Forward primer to sequence PRIM1 stability assay constructs |
| Prim 1 Ex 4 Seq 1R | ATTGTCATGAGGGTCCAGCA | Internal Forward primer to sequence PRIM1 stability assay constructs |
| Prim1 Ex9 seq 2F | CTGGCTGGAGTGGGAGATTA | Internal Forward primer to sequence PRIM1 stability assay constructs |
| Prim1 Ex 9 Seq 2R | TAATCTCCCACTCCAGCCAG | Internal Forward primer to sequence PRIM1 stability assay constructs |
| Q5SDM_C301R_F | GCTCCAGTACCGTTTTCCACG | Forward primer to introduce PRIM1 C301R mutation (c.901 T>C, TGT>CGT) |
| Q5SDM_C301R_R | ATAATCTCCCACTCCAGC | Reverse primer to introduce PRIM1 C301R mutation (c.901 T>C, TGT>CGT) |
| Q5SDM_V35insDGV_F | GGAGTGATAAAGAATTACTTTCAACACCG | Forward primer to introduce PRIM 3aa insertion mutation (p.V35insDGV; c.104_105insTGATGGAGT) |
| Q5SDM_V35insDGV_R | ATCAACTCCACCGTAGTTGAGCC | Reverse primer to introduce PRIM 3aa insertion mutation (p.V35insDGV; c.104_105insTGATGGAGT) |
| BspEI-PRIM1-ex1F | tctaTCCGGACTTACCGTGGCGAGTTCCG | Forward primer to introduce *BspE*I restriction site upstream of *PRIM1* exon 1 for cloning into RHCglo |
| XbaI-PRIM1-intr2R | ctagTCTAGAGAATCAACCCATTCTTGGTTTAC | Reverse primer to to introduce *Xba*I restriction site downstream of *PRIM1* intron 2 for cloning into RHCglo |
| Prim1 RHCglo F | CGAACCACTGAATTCCGCAT | External Forward primer to sequence *PRIM1* minigene clones in RHCglo |
| RHCglo-R primers | GATGCGGCCCTGAAGTTGTTC | External Reverse primer to sequence *PRIM1* minigene clones in RHCglo |
| Prim1 mini seq  F | CATCTACCACGGTGTTGAGC | Internal Forward primer to sequence *PRIM1* minigene clones in RHCglo |
| Prim1 mini seq R | AGTGCTGGGATTATAGGCGT | Internal Reverse primer to sequence *PRIM1* minigene clones in RHCglo plasmid |
| Q5SDM_PRIM1_donor1_F | TACGGTGGAGTTGATGGAGGC | Forward primer to introduce PRIM1 donor splice mutation (c.103+1 G>T) at PRIM1 Ex1/Int1 boundary |
| Q5SDM_PRIM1_donor1_R | GTTGAGCCAGCGATAGTAC | Reverse primer to introduce PRIM1 donor splice mutation (c.103+1 G>T) at PRIM1 Ex1/Int1 boundary |
| RSV5U F | CATTCACCACATTGGTGTGC | Forward primer to sequence minigene cDNA products |
| RTRHC R | GGAGCTTTGCAGCAACAGTAAC | Reverse primer to sequence minigene cDNA products |
| PRI1-C-MX6-F | GAACTGGGTTCAGTGAAAAGAGAACGTGAAGATGATGATGAACCGGCTTCTTTAGATTTCCGGATCCCCGGGTTAATTAAG | Forward primer with 60nt homology (underlined) upstream of *PRI1* stop codon and 21nt to anneal to pFA6a cassette, for C-terminal tagging of *S. cerevisiae* primase catalytic subunit |
| PRI1-C-MX6-R | GCTATAGTAGTCATATATATATATATACACCCTTTTTATTGTTACAAAAAGATTTCACCAGAATTCGAGCTCGTTTAAAC | Reverse primer with 60nt homology (underlined) downstream of *PRI1* stop codon and 20nt to anneal to pFA6a, for C-terminal tagging of *S. cerevisiae* primase catalytic subunit |
| TTEF-F | TCGCCTCGACATCATCTGC | Forward primer out of *TEF* terminator (MX6 cassette) to check *PRI1* C-terminal tagging |
| PRI1-doR | CAGCGGAATGCGTTATCAGC | Reverse primer downstream of *PRI1* stop codon to check *PRI1* C-terminal tagging |
| PRI1-L309-gRNA-F | GATCTCTTTATCCGAAGCTGGATGGTTTTAGAGCTAG | Forward oligonucleotide for cloning gRNA (underlined) targeting *PRI1* near L309, into *Bcl*I/*Swa*I of pML104/pML107 |
| PRI1-L309-gRNA-R | CTAGCTCTAAAACCATCCAGCTTCGGATAAAGA | Reverse oligonucleotide for cloning gRNA targeting *PRI1* near L309, into *Bcl*I/*Swa*I of pML104/pML107 |
| PRI1-PAM-F | TTACGTGAATGTAAGGAAGATCTCGTATTGATGACTCTTTATCCGAAGCTGGATGTcGAAGTTACAAAGCAAACAATTCATTTGTTAAAG | 90nt oligo to introduce silent PAM site mutation V315 (GTG>GTC) into *PRI1*; used with pMAR780/781 |
| PRI1-PAM-R | CTTTAACAAATGAATTGTTTGCTTTGTAACTTCgACATCCAGCTTCGGATAAAGAGTCATCAATACGAGATCTTCCTTACATTCACGTAA | 90nt reverse complement oligo to introduce silent PAM site mutation V315 (GTG>GTC) into *PRI1*; used with pMAR780/781 |
| PRI1-L309C-F | TTACGTGAATGTAAGGAAGATCTCGTATTGATGACT**tgT**TATCCGAAGCTGGATGTcGAAGTTACAAAGCAAACAATTCATTTGTTAAAG | 90nt oligonucleotide to introduce L309C (CTT>**TGT**, equivalent to wildtype residue in human *PRIM1*) and silent PAM site mutation V315 (GTG>GTC) into *PRI1*; used with pMAR780/781 |
| PRI1-L309C-R | CTTTAACAAATGAATTGTTTGCTTTGTAACTTCgACATCCAGCTTCGGATA**Aca**AGTCATCAATACGAGATCTTCCTTACATTCACGTAA | 90nt reverse complement oligo to introduce L309C (CTT>**TGT**, equivalent to wildtype residue in human *PRIM1*) and silent PAM site mutation V315 (GTG>GTC) into *PRI1*; used with pMAR780/781 |
| PRI1-L309R-F | TTACGTGAATGTAAGGAAGATCTCGTATTGATGACT**CgT**TATCCGAAGCTGGATGTcGAAGTTACAAAGCAAACAATTCATTTGTTAAAG | 90nt oligo to repair *PRI1* to introduce L309R (CTT>**CGT**, equivalent to PD mutation in human *PRIM1*) and silent PAM site mutation V315 (GTG>GTC) into *PRI1*; used with pMAR780/781 |
| PRI1-L309R-R | CTTTAACAAATGAATTGTTTGCTTTGTAACTTCgACATCCAGCTTCGGATA**AcG**AGTCATCAATACGAGATCTTCCTTACATTCACGTAA | 90nt reverse complement oligo to repair PRI1 to introduce L309R (CTT>**CGT**, equivalent to PD mutation in human *PRIM1*) and silent PAM site mutation V315 (GTG>GTC) into *PRI1*; used with pMAR780/781 |
| PRI1-F | GAAGATGACCAACATGCTATC | Forward primer to PCR amplify a 382bp product surrounding *PRI1*-L309 for sequencing. Used with PRI1-R |
| PRI1-R | TCTCCATTTCTGTTTGAAGATC | Used with PRI1-F |
| XbaI-PRI1-F | gctctagaccATGACCAATTCAGTAAAGACTAA | Forward primer to introduce XbaI site and Kozak sequence upstream of yeast *PRI1* coding sequence for cloning into pGFP-C-FUS |
| BamHI-PRI1ns-R | aaggatccGAAATCTAAAGAAGCCGGTTC | Reverse primer to introduce *BamH*I site downstream of yeast *PRI1* coding sequence for cloning into pGFP-C-FUS, no stop, in frame with *GFP* |
| PMet25F | GTCGTCAGATACATAGATAC | Forward sequencing primer out of *MET25* promoter from pGFP-C-FUS |
| MET15-F | CTGATATCTTCGGATGCAAGG | Forward primer to amplify 2,652 bp fragment to reconstitute *MET15* in BY4741. Used with MET15-R |
| MET15-R | AACTTTGTTGAATGTTGAGCAAG | Used with MET15-F |
| MET15-upF | GTTTAAGGCGTCAGATTTAGG | Forward primer to check 5' end of *MET15* reconstitution. 220bp fragment with MET15-intR |
| MET15-intR | CAGTTTTCATGAGGATGGCG | Used with MET15-upF |
| MET15-intF | GTATTAGTTGATAGACAATAGTGG | Forward primer to check 3' end of *MET15* reconstitution. 213bp fragment with MET15-doR |
| MET15-doR | CCGCGTTATTAGCACGGTG | Used with MET15-intF |
