# Supplementary material for: PRIM1 deficiency causes a distinctive primordial dwarfism syndrome
Source: Genes Dev. 2020 Nov 1;34(21-22):1520–33. doi: 10.1101/gad.340190.120 (PMC7608753; doi:10.1101/gad.340190.120)
Supplement: Supplemental Material [file supp_gad.340190.120_Supplemental_Table_S10.docx]

**Supplemental Table S10: Plasmids used in this study**

| Plasmid | Description | Reference |
| --- | --- | --- |
| pFA6a-13Myc-HphMX6 | PCR template to add a C-terminal 13xMyc tag using homologous recombination and hygromycin selection. A gift from Jean Beggs | (Spiller et al. 2007) |
| pML104 | Expresses Cas9 and contains guide RNA expression cassette with *Bcl*I-*Swa*I cloning sites for guide sequence cloning. Contains *URA3* marker for yeast transformation. A gift from John Wyrick (Addgene plasmid #67638 ; http://n2t.net/addgene:67638) | (Laughery et al. 2015) |
| pML107 | Expresses Cas9 and contains guide RNA expression cassette with *Bcl*I-*Swa*I cloning sites for guide sequence cloning. Contains *LEU2* marker for yeast transformation. A gift from John Wyrick (Addgene plasmid # 67639 ; http://n2t.net/addgene:67639) | (Laughery et al. 2015) |
| pGFP-C-FUS | Yeast shuttle vector (*CEN6*, *ARSH4*, *URA3*, AmpR) for C-terminal tagging and expression from the methionine regulatable *MET25* promoter. A gift from Jean Beggs | (Niedenthal et al. 1996) |
| RHCglo | Minigene reporter for identification and analysis of cis elements and trans factors affecting pre-mRNA splicing. A gift from Thomas Cooper (Addgene plasmid # 80169 ; http://n2t.net/addgene:80169) | (Singh and Cooper 2006) |
| pmGFP-P2A-K0-P2A-RFP | Double fluorescent stalling reporter K0. A gift from Ramanujan Hegde (Addgene plasmid # 105686 ; http://n2t.net/addgene:105686) | (Juszkiewicz and Hegde 2017) |
| pMAR780 | pML104 with gRNA targeting *S. cerevisiae* *PRI1* near L309. Oligonucleotide duplex of PRI1-L309-gRNA-F and R, cloned using *Swa*I/*Bcl*I | This study |
| pMAR781 | pML107 with gRNA targeting *S. cerevisiae* *PRI1* near L309. Oligonucleotide duplex of PRI1-L309-gRNA-F and R, cloned using *Swa*I/*Bcl*I | This study |
| pMAR782 | pGFP-C-FUS with *S. cerevisiae* *PRI1* with silent PAM site mutation (c.1945G>C) without stop codon, cloned using *Xba*I/*BamH*I (pGFP-C-PRI1) | This study |
| pMAR790 | PCR amplified 1.35 kb fragment of human genomic DNA (chr12:56750975-56752323, hg38) covering *PRIM1* exon1 and exon 2, cloned into RHCglo (*BspE*I/*Xba*I) fusing *PRIM1* exon 1 to RHCglo exon 1. | This study |
| pMAR793 | Donor splice site mutation (c.103+1G>T) introduced into *PRIM1* intron 1 of pMAR790 by SDM | This study |
| pMAR796 | Human C-terminally EGFP-tagged PRIM1 flanked by *Nhe*I and *BspE*I sites (synthesized by IDT) in pUCIDT-AMP GoldenGate | This study |
| pMAR797 | PRIM1-EGFP cloned from pMAR796 (*Nhe*I/*BspE*I) into pmGFP-P2A-K0-P2A-RFP, replacing mGFP | This study |
| pMAR798 | C301R mutation introduced by SDM in pMAR796 | This study |
| pMAR799 | PRIM1-EGFP-C301R cloned from pMAR798 (*Nhe*I/*BspE*I) into pmGFP-P2A-K0-P2A-RFP, replacing mGFP | This study |
| pMAR802 | V35insVDG mutation introduced by SDM in pMAR796 | This study |
| pMAR803 | PRIM1-EGFP- V35insVDG cloned from pMAR802 (*Nhe*I/*BspE*I) into pmGFP-P2A-K0-P2A-RFP, replacing mGFP | This study |
