# Supplementary material for: PRIM1 deficiency causes a distinctive primordial dwarfism syndrome
Source: Genes Dev. 2020 Nov 1;34(21-22):1520–33. doi: 10.1101/gad.340190.120 (PMC7608753; doi:10.1101/gad.340190.120)
Supplement: Supplemental Material [file supp_gad.340190.120_Supplementary_Text_and_Figures.pdf]

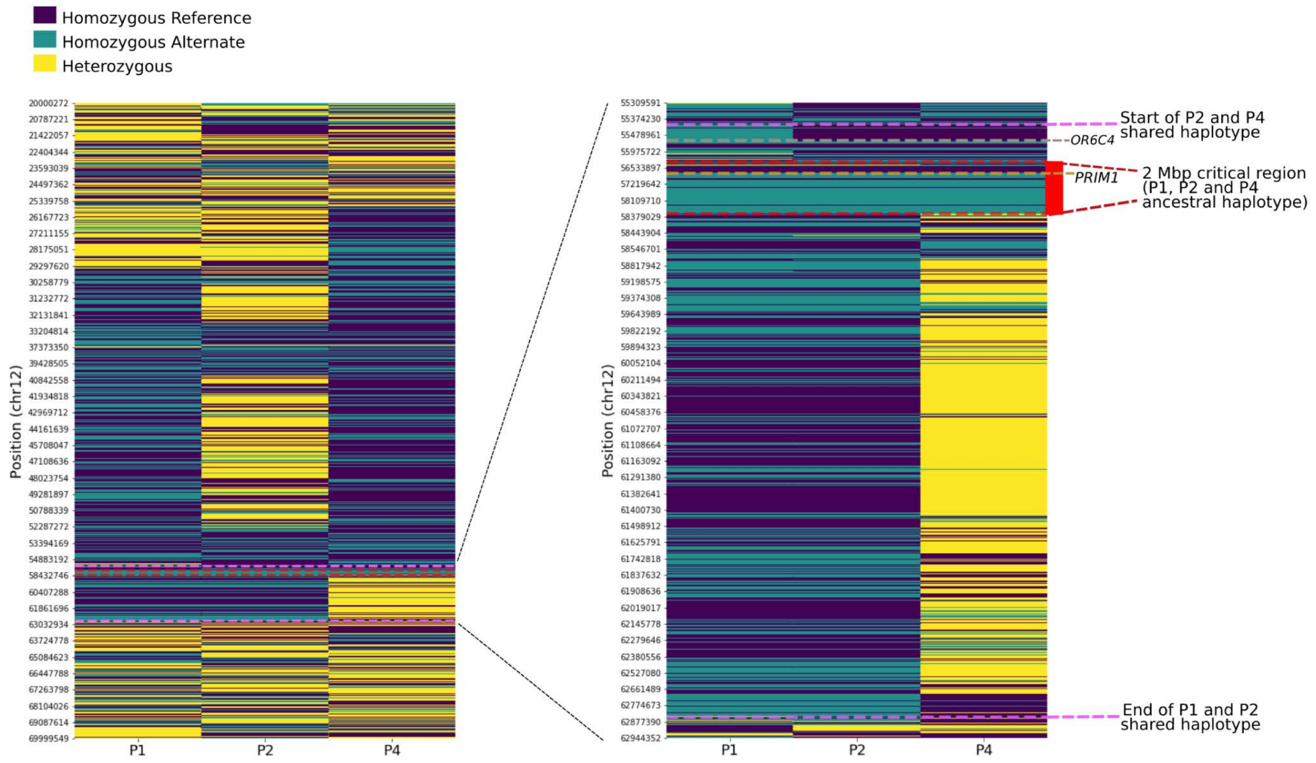

Supplemental Figure S1: Haplotype analysis of Families 1-3. Genotypes from whole genome sequencing of individuals are shown for biallelic single-nucleotide variants. Variants where all individuals share the common allele (AF > 0.5 in gnomAD non-Finnish Europeans) are omitted for clarity. The ancestral haplotype shared by P1, P2 and P4 is marked in red. Pairwise shared haplotypes are marked by pink dotted lines. Details of genotypes are available in Supplemental Table S7.

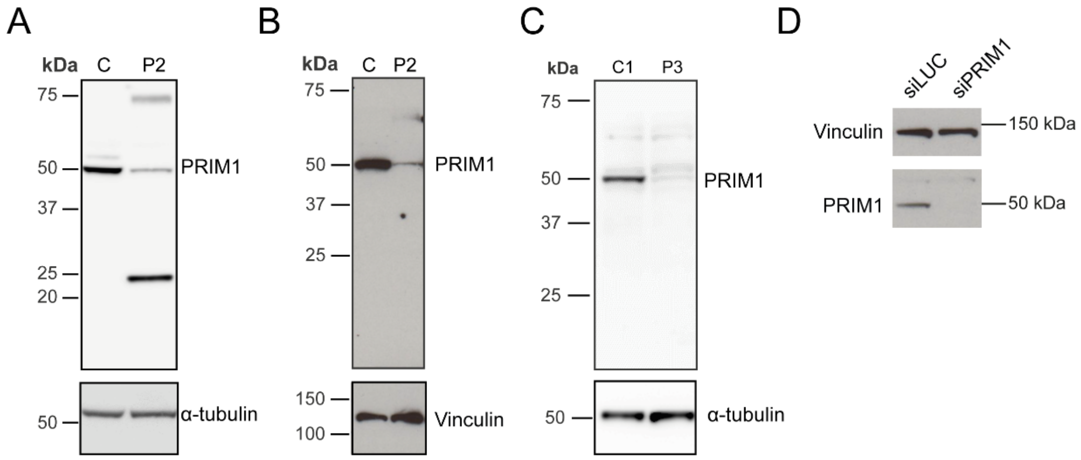

Supplemental Figure S2. **PRIM1 immunoblots (20-75 kDa)**.

(A) Corresponds to Figure 2D, with additional bands seen in P2 at 75 kDa and 25 kDa.

(B) Independent experiment with cell lysates from C and P2 in which additional bands at 25 kDa not evident.

(C) Corresponds to Figure 2D. No additional band visible at 25 kDa.

(D) Validation of monoclonal antibody (8G10) with siRNA to *PRIM1* in HeLa cells. siLUC, luciferase negative control. siPRIM1, siRNA targeting *PRIM1* transcript. Vinculin used as a loading control.

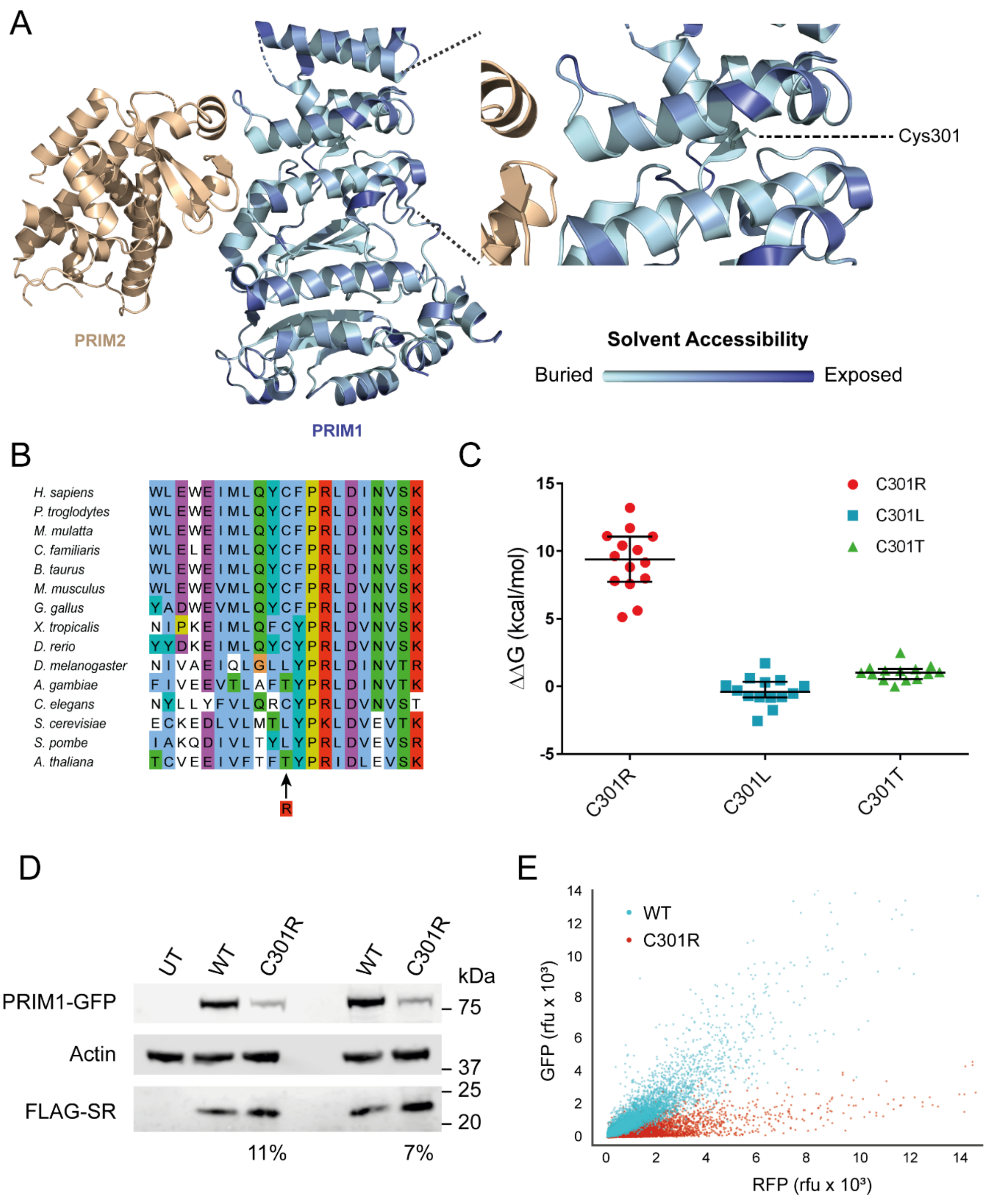

Supplemental Figure S3: Predicted destabilisation of PRIM1 by the C301R substitution.
(A) Cysteine 301 lies in a buried hydrophobic region in PRIM1. DNA primase dimer crystal structure (PDB: 4BPU). PRIM1 residues shaded according to solvent accessibility. Cysteine 301, substituted to arginine in P5, lies in a buried hydrophobic region.

(B) Cys301 is evolutionarily conserved in vertebrates. Neither arginine or other large or charged amino acids are observed at this position in other species.

(C) The C301R substitution observed in P5 is predicted to lead to destabilisation of all available PRIM1 crystal structures. In contrast, substitution with leucine or threonine, as observed in some

orthologous proteins, is not predicted to lead to destabilisation. Data points, predicted changes in free-energy (ΔΔG) from FoldX plotted for each of 14 available crystal structures.

(D) Immunoblotting of RPE1 cells transfected with dual reporter constructs (as described in Figure 3B and Materials and Methods) confirms production of PRIM1-GFP and FLAG-SR at expected molecular weights and shows reduced protein levels for the C301R variant. n=2 experiments shown. C301R protein levels relative to WT after normalization to actin loading control indicated underneath the blot. UT, untransfected; WT, wild-type, C301R, p.Cys301Arg mutant.

(E) GFP-RFP scatter for wild-type and C301R reporter constructs from the same transfection as (D).

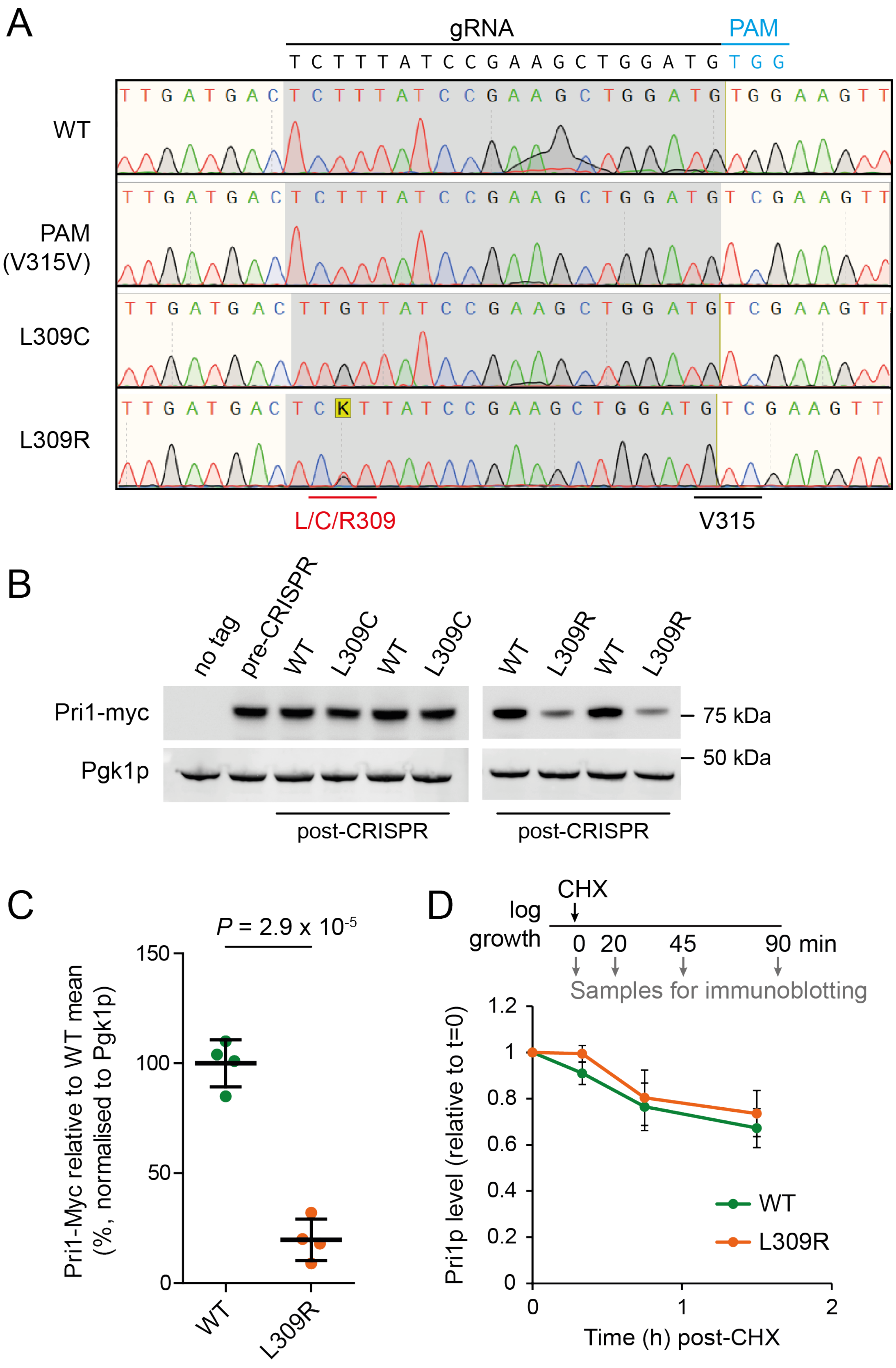
A
gRNA
PAM
T C T T T A T C C G A A G C T G G A T G T G G
WT
PAM (V315V)
L309C
L309R
L/C/R309
V315
B
no tag
pre-CRISPR
WT
L309C
WT
L309C
WT
L309R
WT
L309R
Pri1-myc
Pgk1p
75 kDa
50 kDa
post-CRISPR
post-CRISPR
C
P = 2.9 x 10-5
Pri1-Myc relative to WT mean (%, normalised to Pgk1p)
150
100
50
0
WT
L309R
D
log growth
CHX
0
20
45
90 min
Samples for immunoblotting
Pri1p level (relative to t=0)
1.2
1
0.8
0.6
0.4
0.2
0
WT
L309R
0
1
2
Time (h) post-CHX

Supplemental Figure S4. The L309R substitution causes reduced levels but not an increased rate of degradation of the Primase catalytic subunit Pri1p in budding yeast.

(A) Sanger sequencing confirms introduction of mutations in *S. cerevisiae PRI1* by CRISPR genome editing. Traces are displayed for wild-type *PRI1* and *PRI1* with the following changes: a silent PAM site mutation only (V315V), or in combination with L309C or L309R missense mutations. For L309R a GFP-tagged wild-type copy of *PRI1* (with the silent PAM site mutation only) is also present on a centromeric plasmid to maintain viability (see Supplemental Fig. S5).

(B) Substitution of leucine with arginine at codon 309 (L309R, equivalent to the PRIM1-C301R patient mutation) in *S. cerevisiae* Pri1p causes significantly reduced protein levels, whereas the L309C change (substituting leucine with cysteine, the equivalent residue in wild-type human PRIM1) has no effect. Immunoblotting for non-Myc-tagged (no tag), a Pri1 Myc-tagged (pre-CRISPR), and two independent strains with a silent PAM site mutation (WT) or with the L309C mutation introduced by CRISPR all show equal levels of Pri1p when expressed from the endogenous locus, whereas independent strains expressing pri1-L309R show significantly reduced Pri1 protein. Protein extracts for L309C and controls prepared from strains grown in YPD, for L309R and controls strains were grown in SD-Ura-Met medium (to allow expression of Pri1-GFP from the "rescue" plasmid). Pgk1p, loading control.

(C) Quantification of Pri1-myc immunoblotting in B, normalised to the Pgk1p loading control. L309R Pri1-myc protein is significantly reduced (20±9%, mean ± SD). Data points, values for individual samples normalised to the average level in PRI1 WT strains, horizontal line and error bars: mean ± SD. *P*-values, 2-sided unpaired t-test with unequal variances.

(D) Cycloheximide chase experiment, performed with yeast grown in log phase in SD-Ura-Met medium, shows similar degradation dynamics for WT and L309R Myc-tagged Pri1 protein. Data points and error bars (mean ± SD) for experiments with 3 independent WT (MRY205, 223 and 224) and 3 independent pri-L309R strains (MRY206, 227 and 228). Intensity of Pri1-myc bands after immunoblotting was normalised to t=0 values for each experiment.

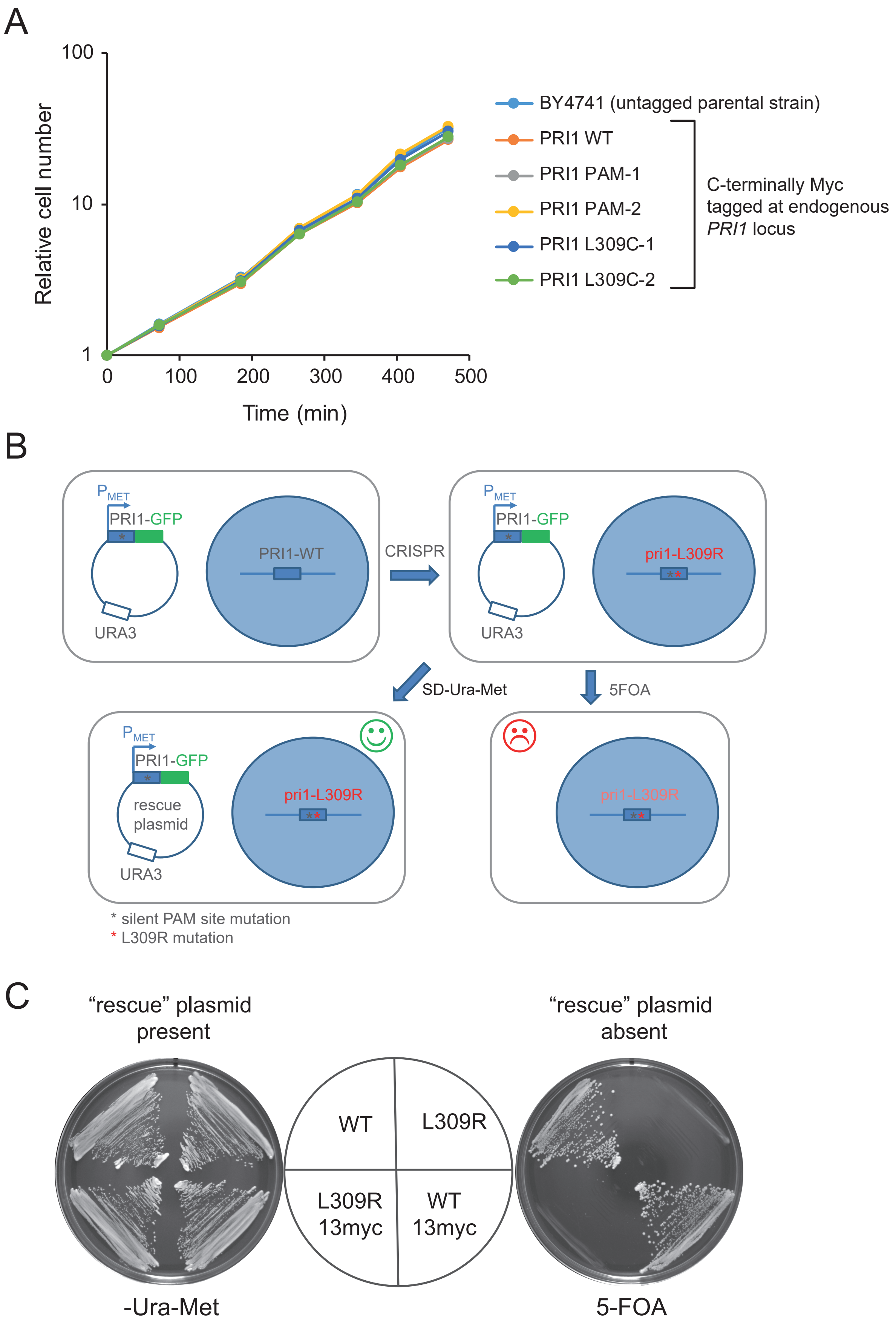
A
100
10
1
Relative cell number
0
100
200
300
400
500
Time (min)
BY4741 (untagged parental strain)
PRI1 WT
PRI1 PAM-1
PRI1 PAM-2
PRI1 L309C-1
PRI1 L309C-2
C-terminally Myc tagged at endogenous *PRI1* locus
B
PMET
PRI1-GFP
URA3
PRI1-WT
CRISPR
pri1-L309R
SD-Ura-Met
5FOA
rescue plasmid
* silent PAM site mutation
* L309R mutation
C
"rescue" plasmid present
"rescue" plasmid absent
WT
L309R
L309R 13myc
WT 13myc
-Ura-Met
5-FOA

Supplemental Figure S5. The orthologous L309R mutation, corresponding to the PRIM1-C301R patient mutation, is non-viable in budding yeast.

Given functional conservation between yeast and human PRIM1, and to investigate the cellular consequences of the C301R missense mutation we introduced orthologous variants at this codon into *S. cerevisiae*. Initially, we attempted to introduce both the L309C and L309R mutations into budding yeast Pri1, changing the equivalent residue to that of wild-type human PRIM1 or patient PRIM1 respectively. Despite several attempts, no colonies were found to carry the L309R mutation (0 out of 32 colonies), although 94% (30) of these did have the silent PAM site mutation. In contrast, 50% of colonies (8 out of 16) carried the L309C mutation, all of which showed normal growth (**A**). This suggested that the L309R mutation but not the L309C mutation may impact on viability. Lower colony numbers for each L309R CRISPR editing attempt (48±8% colonies compared to L309C, n=4 independent attempts) were consistent with this. Therefore, we instead employed a plasmid shuffle method (**B, C**), which confirmed lethality of this mutation in budding yeast.

(A) Growth is not affected by C-terminal Myc-tag, PAM-site mutant or introduction of the L309C substitution. Normal growth of independent yeast strains with C-terminally Myc-tagged Pri1p-L309C (MRY203/204) or silent PAM site mutations (PAM; MRY201/202), compared to non-tagged wild-type (BY4741) and Myc-tagged parental strains (WT, MRY175). Relative OD_{600} values plotted for yeast grown in YPD medium at 30°C and kept in log phase (OD_{600} = 0.2-0.8), by diluting in fresh medium when necessary.

(B) Diagram outlining methodology for introducing the L309R mutation by CRISPR at the genomic *PRI1* locus. Viability of this strain is maintained by expression of an episomal copy of the wild-type *PRI1* gene, while the mutation is introduced at the endogenous locus by CRISPR. The episomal gene remains unedited due to a silent mutation at the PAM site preventing editing. Once the L309R mutation has been confirmed to be present, viability of the L309R mutation is tested by plating on 5-FOA medium, which leads to loss of the WT-PRI1 (“recue”) plasmid due to negative selection for the *URA3* selection cassette.

(C) Yeast strains expressing Pri1-L309R from the endogenous genetic locus and WT-PRI1 from a centromeric *URA3* vector, grow normally on SD-Ura-Met plates, but cannot survive on 5-FOA containing medium, regardless of the presence or absence of a C-terminal Myc-tag. On 5-FOA medium only the genomic copy of *PRI1* is expressed, due to negative selection against the *URA3* vector. Colonies with the L309R mutation (MRY200, MRY206) or with a silent PAM site mutation only (WT; MRY199, MRY205) were streaked out after CRISPR-mediated genome editing of non-tagged (BY4741) and 13myc-tagged Pri1p yeast (MRY175), and grown at 30°C. The same results were obtained for all independent L309R and control strains (Supplemental Table S11).

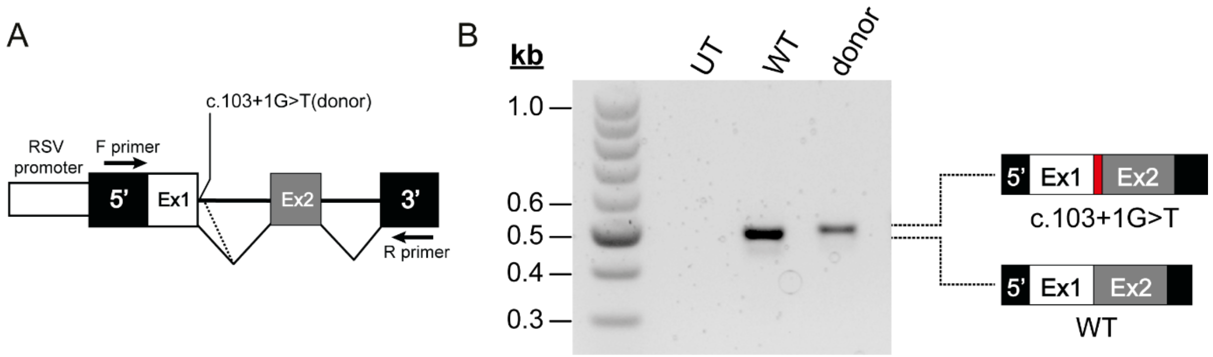

Supplemental Figure S6: Minigene splicing assay of the c.103+1G>T variant.

(A) Schematic of minigene assay. Genomic DNA spanning exon1 (Ex1) and exon 2 (Ex2) of *PRIM1* was cloned into minigene reporter construct and the c.103+1G>T variant introduced by site-directed mutagenesis. Arrows, PCR primers used to assay splicing from cDNA following transfection. Dotted line indicates alternate splicing from the cryptic splice donor (Fig. 3E).

(B) Agarose gel electrophoresis of PCR products generated from the minigene assay. UT, untransfected; WT, wild-type construct; donor, c.103+1G>T variant construct. Right, Schematic of the minigene construct mRNA after splicing. Nine nucleotides of intronic sequence are inserted (red) relative to the wild-type construct (Figure 3G).

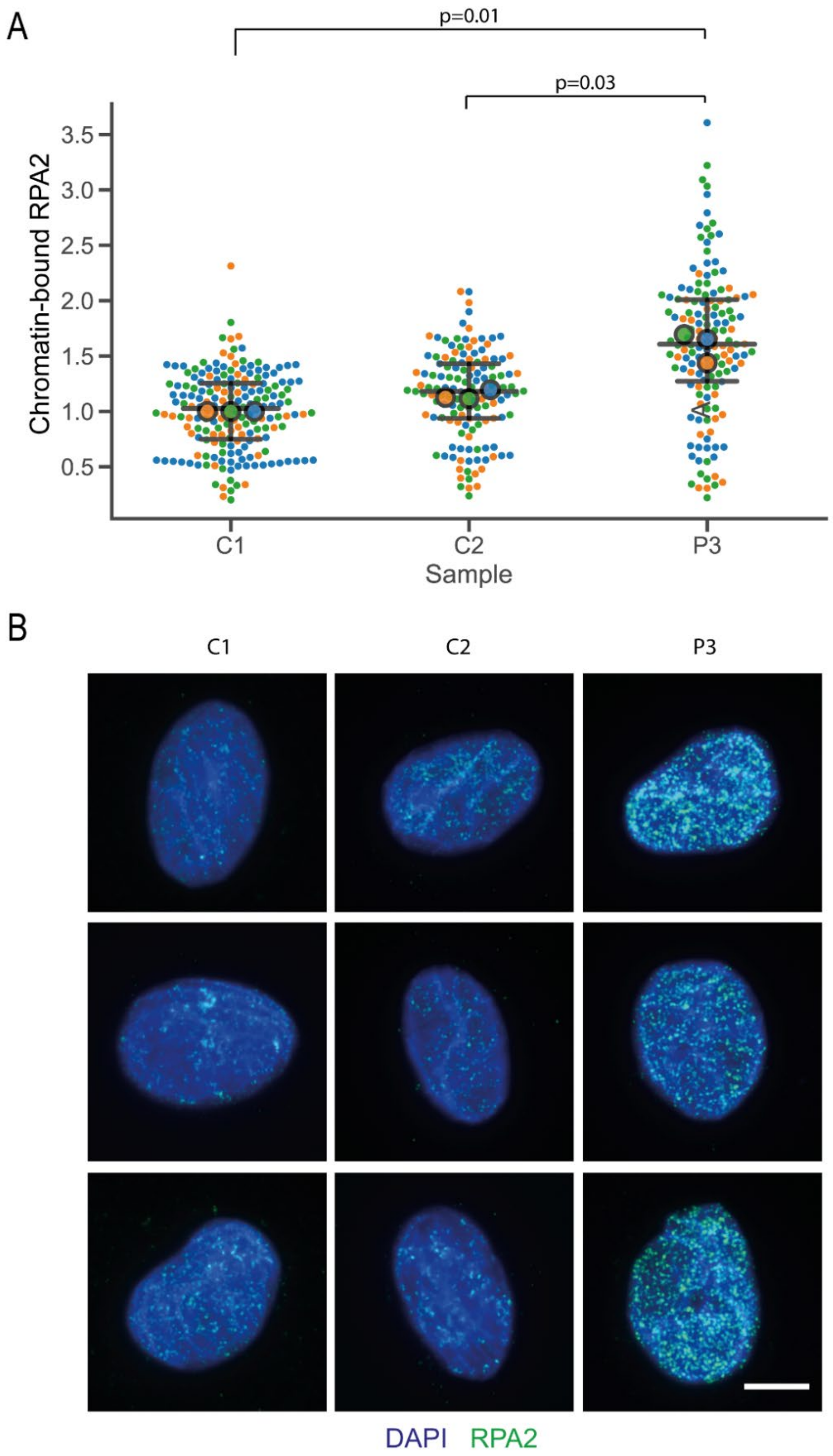

Supplemental Figure S7: Chromatin-bound RPA levels are increased in PRIM1 deficient fibroblasts. (A) Mean RPA2 fluorescence calculated from C1, C2 and P3 cells normalized to mean values of C1 for each experiment. n=3 experiments. Data points coloured by experiment; filled circles, mean values for each replicate; bars, median and interquartile range (for all values); P-values, repeat-measures ANOVA on pre-normalized mean nuclear RPA2 intensities with Tukey multiple comparison test.

(B) Representative immunofluorescence images. Blue, DAPI; green, RPA2. Scale bar, 5 µm.

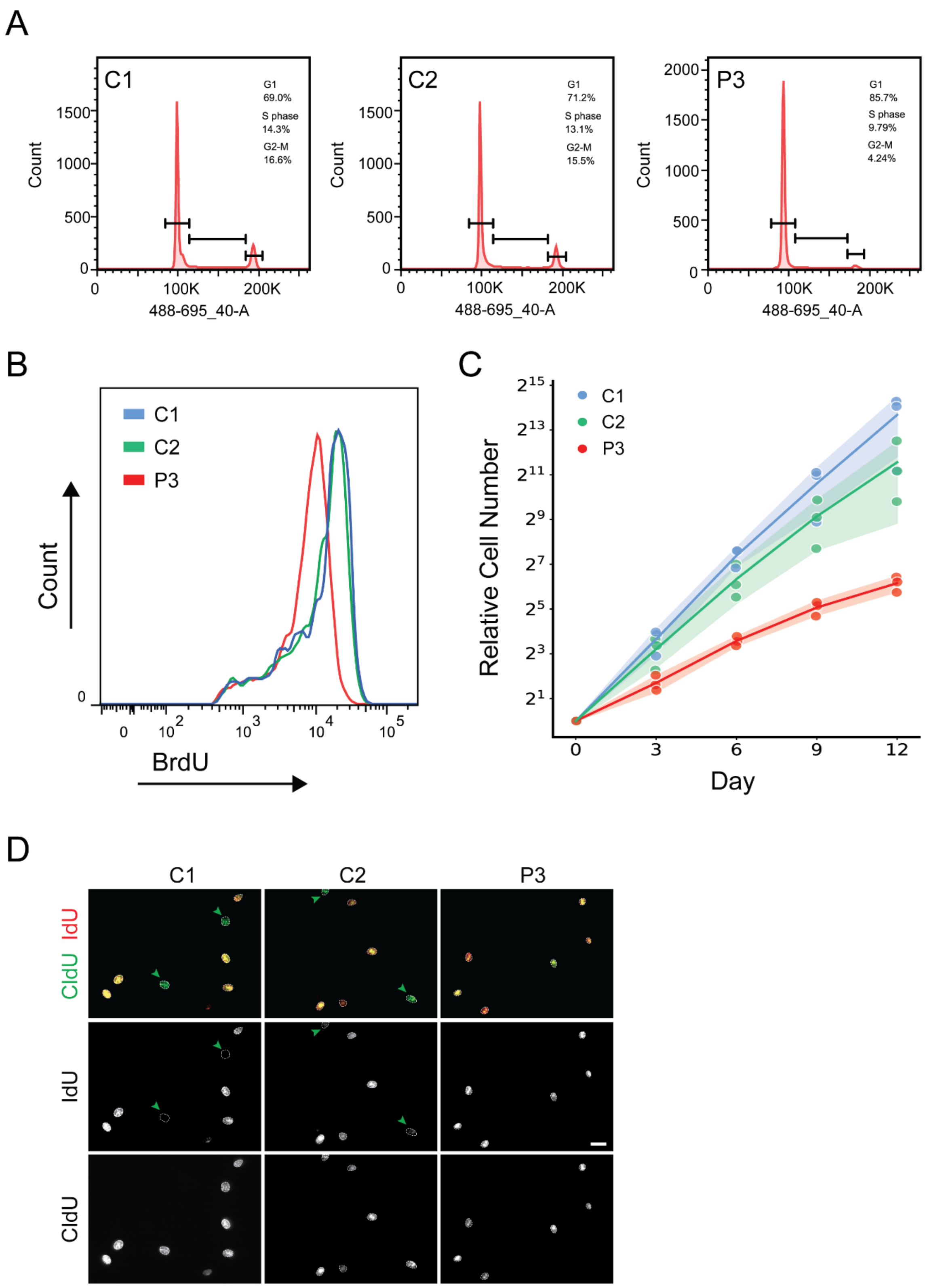

Supplemental Figure S8: Cell cycle analysis of P3 fibroblasts.

(A) DNA content histograms for C1, C2 and P3 cells replotted from the representative flow cytometry experiment shown in Figure 4F.

(B) BrdU mean fluorescence intensity (MFI) histogram, representative experiment of n=3 experiments plotted in Figure 4G.

(C) Time course of relative cell number plotted from passage-matched primary fibroblast cell lines. Shaded areas indicate ± one standard deviation for each sample at each timepoint. n=3 experiments. Cell doubling times plotted in Fig. 4A derived from this data.

(D) Representative immunofluorescence images of fibroblasts from the CldU/IdU double-labelling experiment from Figure 4B,C. Scale bar, 20 µm. Arrow heads indicate cells exiting S-phase between CldU and IdU pulses.

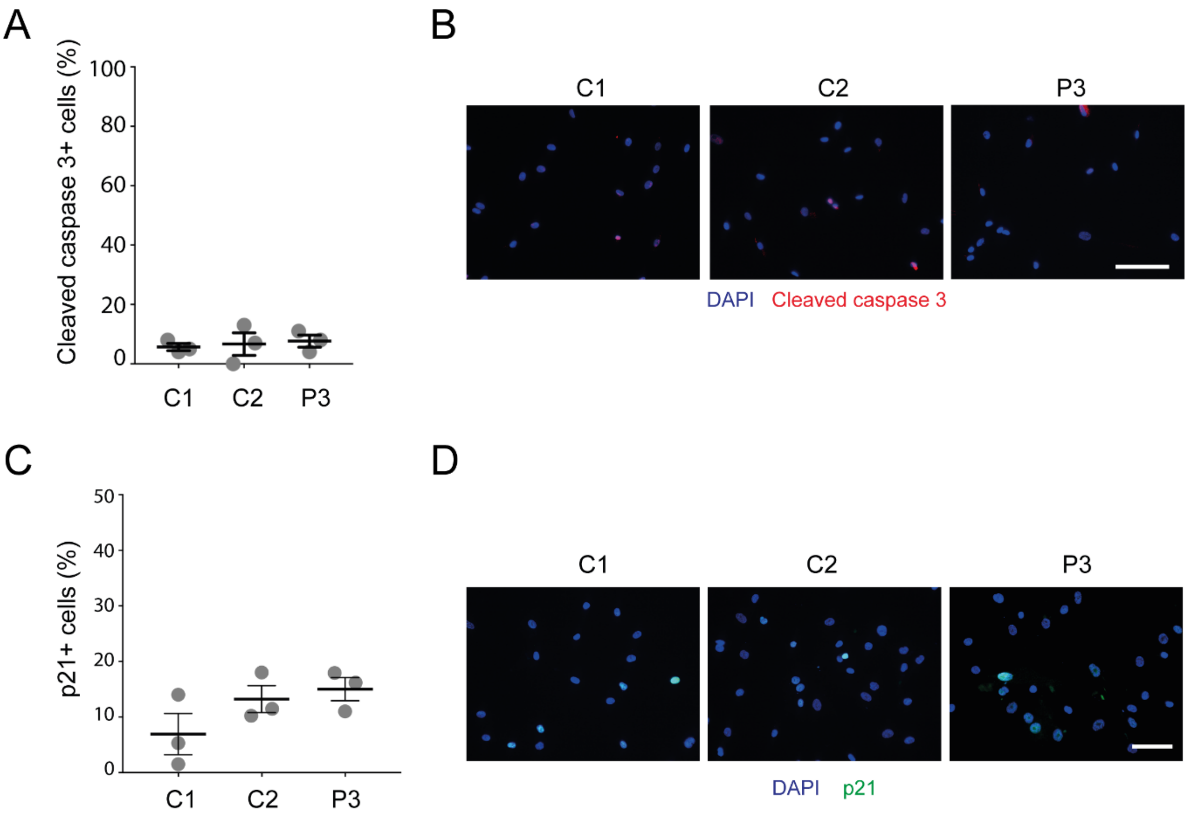

Supplemental Figure S9: Proportion of apoptotic or p21 positive cells is not increased in P3 fibroblasts compared with controls.

(A) Percentage cleaved caspase 3 positive cells from C1, C2 and P3 fibroblasts. n=3 expts. One-way ANOVA P=0.4.

(B) Representative immunofluorescence images of cleaved caspase 3 staining. Scale bar, 50 µm.

(C) Percentage p21 positive cells from C1, C2 and P3 fibroblasts. n=3 expts. One-way ANOVA P=0.2.

(D) Representative immunofluorescence images of p21 staining. Scale bar, 50 µm.

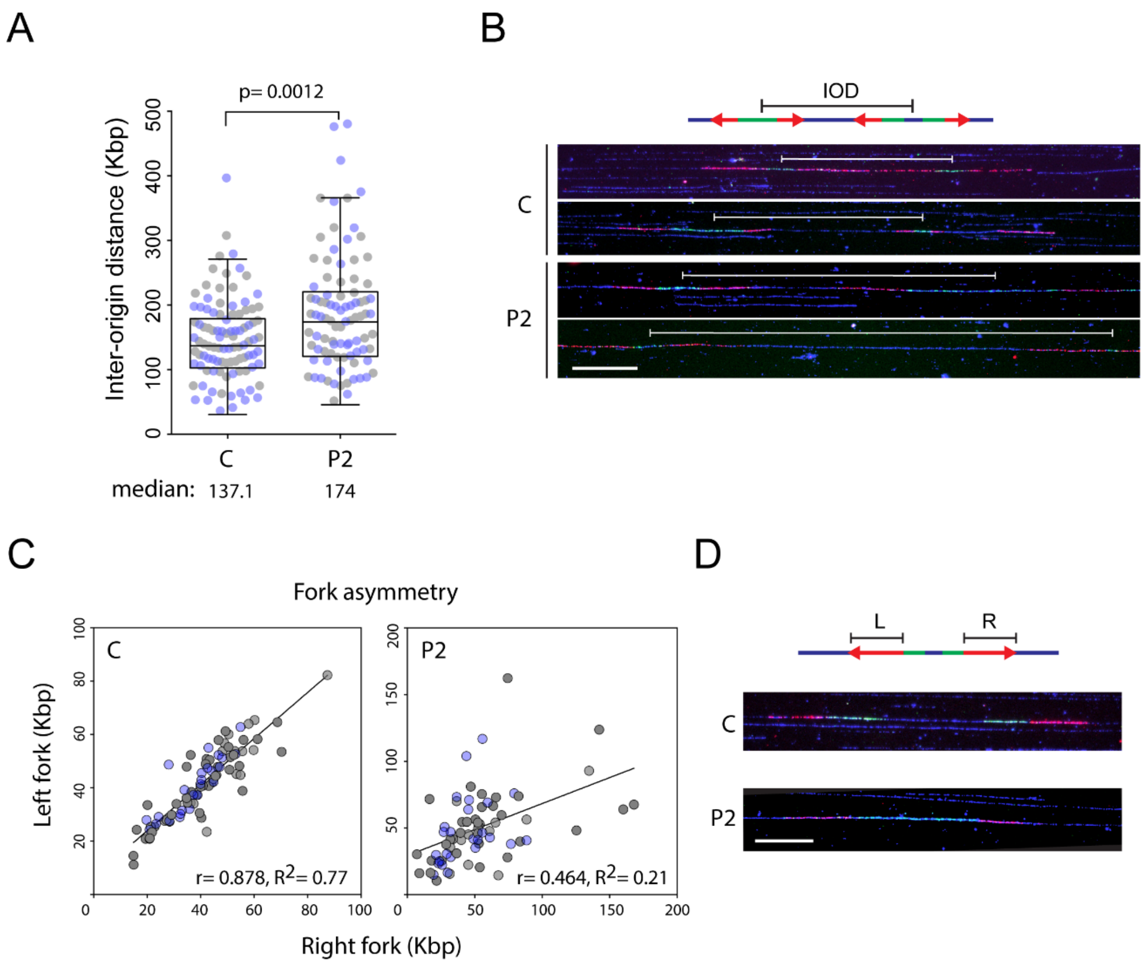

Supplemental Figure S10: Increased inter-origin distances (IODs) and fork-asymmetry in P2 lymphoblastoid cells.

(A) Individual data points coloured per experiment. n=2 expts. Boxplots, Tukey distribution; P-values, Mann–Whitney U test.

(B) Representative combing images, indicating IODs Scale bar, 25 µm.

(C) Fork ratio scatter plots. r, Pearson correlation coefficient.

(D) Representative images of bi-directional forks. Scale bar, 20 µm.

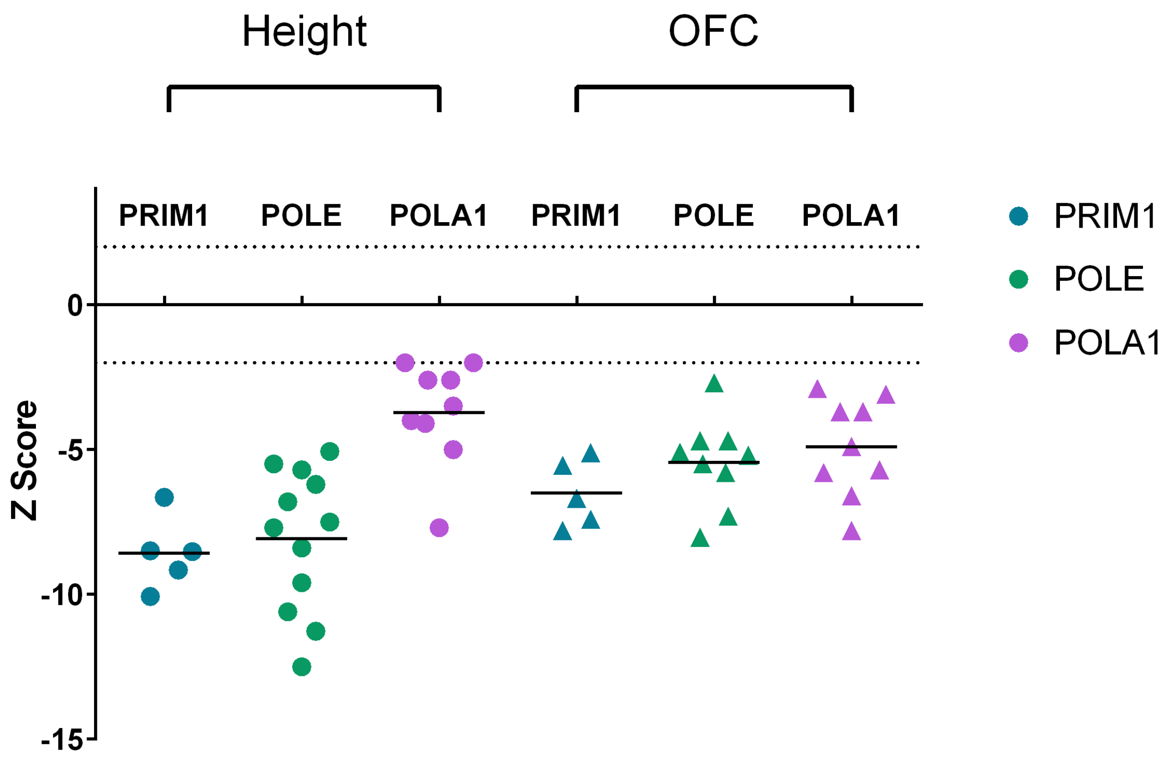

Supplemental Figure S11: Growth parameters of individuals with *POLA1*, *POLE* and *PRIM1* associated growth disorders. Z scores for postnatal height and occipitofrontal circumference (OFC) are plotted for individuals with PRIM1 deficiency (this study) and previously published individuals with POLE (Logan et al. 2018) or POLA1 (Van Esch et al. 2019) associated disorders.

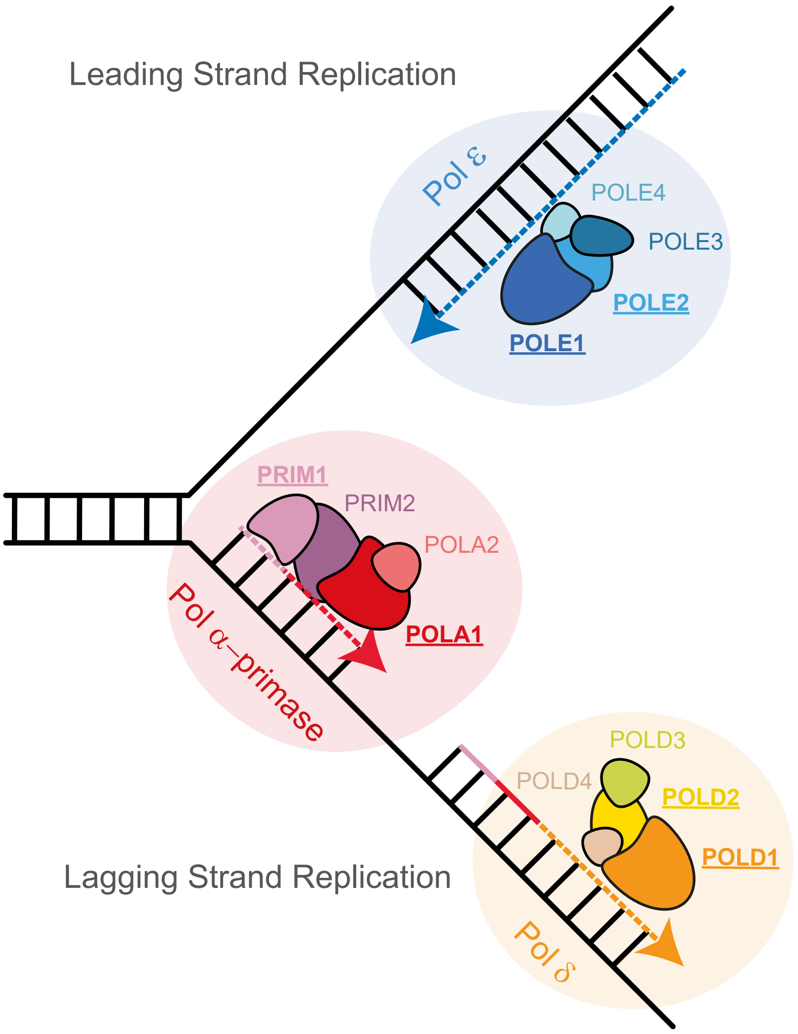

Supplemental Figure S12. The DNA replication fork and microcephalic primordial dwarfism (MPD) spectrum disorders. Schematic of the DNA replication fork. Pol α-primase initiates DNA replication at replication origins by synthesis of an RNA primer by PRIM1, which is extended by the DNA synthesising catalytic subunit of Pol α, POLA1. Pol δ continues DNA synthesis after the synthesis of the short RNA-DNA hybrid primer. On the leading strand Pol ε takes over from Pol δ to perform the bulk of leading strand DNA synthesis. Subunits of the polymerases with variants identified as causing MPD disorders are underlined.

Supplemental Material and Methods

Relatedness and Inbreeding Coefficient Calculation

Cohort WGS variant data were filtered to include only SNVs. Variants that were rare (allele frequency < 1 % in all gnomAD population groupings), below the VQSR truth sensitivity threshold of 99.9% or overlapping low-complexity or segmentally duplicated regions were removed. Inbreeding coefficients were calculated using vcftools (Danecek et al. 2011) '--het' option. Relatedness was calculated using the KING algorithm (Manichaikul et al. 2010) also with vcftools, using the '--relatedness2' option. A minimum genotype quality score cutoff of 20 (--minGQ 20) was set for both vcftools commands.

Prediction of PRIM1 Substitutions on Protein Stability

Fourteen crystal structures of PRIM1 were obtained from PDBe (4BPU, 4BPW, 4BPX, 4LIK, 4LIL, 4MHQ, 4RR2, 5EXR, 6R4S, 6R4T, 6R4U, 6R5D, 6R5E and 6RB4). Each structure was converted using the FoldX (v5) RepairPDB command with default settings to find the energy minima for each structure. Each repaired PDB file was then analysed using the FoldX BuildModel command to assess the free energy change of the C301R, C301L and C301T substitutions with five replicates (--numberOfRuns=5) and the mean free energy change calculated for each substitution. Visualisation and solvent accessibility analysis was performed using pymol (Schrödinger 2015).

Multiple Sequence Alignments

Multiple sequence alignments of PRIM1 orthologs were generated using Clustal (Larkin et al. 2007) and formatted using Jalview (Waterhouse et al. 2009).

Yeast strains, media and growth conditions

Standard yeast culture media were used: Yeast extract Peptone Dextrose (YPD: 10 g/l yeast extract, 20 g/l bactopeptone, 20 g/l dextrose, 20 g/l agar), Synthetic Defined (SD: 6.7 g/l yeast nitrogen base

without amino acids, complete supplement single/double dropout mixture (Formedium), 20 g/l dextrose, 20 g/l agar) and Synthetic Complete (SC: as SD, but with complete supplement mixture, Formedium). Where specified medium was supplemented with hygromycin B (Hyg, 300 mg/l; Calbiochem) or 5-fluoroorotic acid (5-FOA, 1 g/l, Formedium). *S. cerevisiae* cultures and plates were incubated at 30°C, unless otherwise specified. All strains used in this study were isogenic with BY4741 (Brachmann et al. 1998), and are listed in Supplemental Table S11. In MRY175, the endogenous *PRI1* gene was C-terminally tagged with a 13myc tag, using hygromycin resistance for selection(Spiller et al. 2007), making use of a PCR product with 60 bp homology arms. To allow expression of Pri1-GFP from the *MET25* promoter on pMAR782 by growing strains on SD medium without methionine, BY4741 and MRY175 were complemented with a 2.65 kbp PCR fragment (S288C genomic DNA used as template) covering *MET15* (a.k.a. *MET17*/*MET25*), generating MRY215 and MRY216 respectively. Point mutations were introduced into *PRI1* by CRISPR, using previously described methods (Laughery et al. 2015) along with pMAR780 or pMAR781 and annealed oligonucleotides (Supplemental Table S9). Strains with the silent V315V (c.1945G>C) PAM site mutation (MRY199, 201, 202, 205, 223, 224 and 225) were by-products of CRISPR/Cas9-mediated genome editing to incorporate the L309C (MRY203, 204) or L309R (MRY200, 206, 226, 227, 228) mutations. Insertions were confirmed by PCR, point mutations were confirmed by Sanger sequencing.

siRNA

HeLa cells (1.5 x 10^5) were plated in a 6-well plate 24 h prior to transfection. Cells were transfected with siGENOME Human PRIM1 (5557) siRNA – SMARTpool oligonucleotides (Horizon Discovery, M-020200-02-0005) in Opti-MEM Reduced Serum Medium using Oligofectamine (Life Technologies) as per manufacturer's instructions. A custom siRNA targetting Luciferase (CUUACGCUGAGUACUUCGA) was used as a control. Transfected cells were lysed and analysed by immunoblotting 48 h post-transfection.

Immunoblotting of Yeast Whole Cell Extracts

To prepare whole cell protein extracts from yeast, a previously described method was used (Volland et al. 1994). Briefly, 3 OD_{600} units of overnight yeast culture were pelleted, cells lysed in 0.2 M NaOH and proteins precipitated with 5% TCA. Proteins were pelleted and resuspended in 35 µl dissociation buffer (0.1 M Tris-HCl pH 6.8, 4 mM EDTA, 4% SDS, 20% (v/v) glycerol, 2% (v/v) ß-mercaptoethanol, 0.02% (w/v) bromophenol blue) and the solution neutralised with 15 µl of 1 M Tris base. Samples were heated at 95˚C for 10 min before separation on 4-12 % NuPAGE gels (Life Technologies). Immunodetection of proteins transferred to PVDF was performed using the following primary antibodies: anti-Myc (Cell Signaling, 9B11, 1:2,000) and anti-PGK1 antibody (Thermo Fisher Scientific, 22C5D8, 1:2,000; kindly provided by Aziz El Hage). Finally, detection was performed using Amersham ECL Prime Western Blotting Detection Reagent on the ImageQuantLAS4000 (GE Healthcare Life Sciences), or the Odyssey CLx Imaging System (LI-COR Biosciences). Quantifications were performed using ImageQuant TL 7.0 and Image Studio Lite 5.2, respectively.

Cycloheximide chase

Cycloheximide chase experiments were performed using a previously described method (Buchanan et al. 2016) with minor modifications. Briefly, yeast strains were grown in SD-Ura-Met medium at 30˚C until they reached mid log phase (OD_{600} 0.6-0.8), cells pelleted and resuspended in fresh medium at 2.5 OD units per ml. Cycloheximide was added at 250 µg/ml (Alfa Aesar, J66901; stock at 20 mg/ml in ethanol), cells grown at 30˚C with continuous agitation (250 rpm) and 950 µl of cells collected at the appropriate time points. Cell suspensions were mixed with 50 µl ice-cold 20x stop mix (200 mM sodium azide, 5 mg/ml BSA), temporarily stored on ice and processed for immunoblotting. Cell pellets were resuspended in 100 µl distilled water, mixed with 100 µl 0.2 M NaOH and incubated at room temperature for 5 min. Cells were then pelleted, resuspended in 50 µl SDS-loading buffer (50 mM Tris.HCl pH6.8, 2% (w/v) SDS, 10% (v/v) glycerol, 0.63 M β-

mercaptoethanol, bromophenol blue), heated for 10 min at 95˚C and 4 µl of each sample used for SDS-PAGE and immunoblotting as described above.

Supplemental Note: Case Reports

P1 – female

6th child of 3rd cousin Hungarian parents. Brother also likely affected.

Born small for gestational age at 30 weeks gestation, weight 740 g, length 34 cm, OFC 25 cm. Ventilated for first 4 days of life, then nasal CPAP for a further 8 days.

At 4.5 months of age, severe growth retardation, consistent with severe primordial dwarfism, was evident: weight 1880g; length 40 cm; OFC 30 cm. While there was an absolute microcephaly, short stature was more extreme, leading to an appearance of relative macrocephaly. Examination findings were of a broad high forehead, a late-closing fontanelle, blepharophimosis, small eyes, low-set, underdeveloped ears and a flat nasal bridge. The mouth was straight and horizontal, with overreaching of the upper lip. She had a generalized hypotonia, along with small, lax hands and feet with puffy backs and tapering fingers. Absence of subcutaneous fat was also noted.

Developmentally, she was attentive, but did not attain any motor milestones.

From 5 months of age, she experienced persisting lower-airway obstructive symptoms and chronic cough unresponsive to therapy that progressed to end-stage acute respiratory distress syndrome (ARDS), with Chest X-ray demonstrating a homogenous, increased opacification of the lungs. Death occurred at 7 months of age, due to RSV and parainfluenza 2 bronchiolitis (despite initiation of passive RSV immunization one month prior).

Lymphopenia was evident during her life, with an absolute lymphocyte count 1.8 x 10^9/L (Ref.: 2.2-8.0 x 10^9), as well as a recurrent thrombocytopenia dropping as low as 20 x 10^9/L, (Ref.: 150-620 x 10^9) along with anemia (lowest hemoglobin, 55 g/L, Ref.: 90-135 g/L), requiring occasional platelet and red blood cell transfusion. At 6 months of age, serum IgG, IgA and IgM were undetectable: IgG: <0.75 g/L (Ref.: 2.32-14.11 g/L), IgA: <0.1g/L (Ref.: 0-0.83 g/L), IgM: 0.2g/L (Ref.: 0-1.45 g/L), leading to regular intravenous immunoglobulin (IVIG) supplementation. Total white blood count and neutrophil count normal, respectively 6.2 x 10^9/L and 2.8 x 10^9/L (Ref.: 6.0-13.4 x 10^9/L and Ref: 1.4-9x10^9/L) Lymphocyte subset percentages normal.

Liver function tests were abnormal, alkaline phosphatase raised at 1612 U/L (Ref.: 80-440 U/L), with LDH, GOT, GPT and GGT also elevated. LDH 1402 U/L (Ref: <650 U/L), GOT: 143 U/L (Ref: <40 U/L), GPT 71 U/L (Ref: <40 U/L), GGT: 105 U/L (Ref: <40U/L).

Cardiological assessment demonstrated a 3 mm persistent foramen ovale alongside a patent ductus. Ophthalmology: tortious retinal vessels and macular atrophy by age 6 months.

Her brother (5th child in the family), was phenotypically similar, dying as a neonate. He similarly had proportionate, severe growth retardation (birth weight -2.7 SD, 1490 g, 35 wks), low-set and malformed ears, relative macrocephaly, hypoplastic mandible, and a patent ductus arteriosus. As well, cholestasis, mitral + tricuspid valve insufficiency, and hypocalcified vertebrae were reported.

P2 -female

Consanguinity or ethnicity unknown; lives in a children's home.

Born with significant intra-uterine growth retardation at 38 weeks, birth weight 1470 g, length 40 cm, OFC 32 cm. Prophylactic antibiotics given perinatally and a neonatal anemia corrected by transfusion. Cardiac ultrasound demonstrated a patent foramen ovale and mild aortic arch hypoplasia (the latter not evident on later scan in childhood).

A severe sagittal craniosynostosis resulted in marked scaphocephaly and dilated lateral ventricles. This necessitated skull reconstruction surgery at 4 months, and ventriculo-peritoneal shunt at 7 months.

Morphologically, the patient had a high broad forehead with frontal bossing and flat supraorbital ridges. Skull veins were visible through the skin. Facially she had blepharophimosis, small eyes, epicanthus inversus, a broad flat nasal bridge, anteverted nares, a long philtrum and a large and horizontal mouth. The ears were low-set and malformed – aside from the tragi and helices, no other folding could be distinguished. The hands and feet were small with tapering fingers. Skin folds were visible on the limbs, joints were lax, and subcutaneous fat tissue underdeveloped.

Developmentally, she was significantly delayed, at 23 months of age being able to wave "bye", babble, clap hands and roll over. At approaching 5 years of age she was able to sit unsupported and speak 3-4 words. By 5 years growth remained markedly impaired with extreme microcephalic dwarfism evident: weight 6095 g, length 71 cm, OFC 41 cm.

Respiratory involvement appeared minimal, with only increased perihilar density on chest X-ray noted.

Infections accompanied by fever and raised C-reactive protein occurred on several occasions. These included an episode of ventriculitis treated with long-term antibiotics and neurosurgical revision; another due to Rotavirus and Clostridium difficile enteritis. During an episode of antibiotic-treated sepsis aspirin therapy was initiated for a transient thrombocytosis (thrombocytes 1154 x 10^9/L (Ref.: 150-620 x 10^9/L)).

Immunologically, IgG hypogammaglobulinemia was evident. At 21 months, IgG: 3.5 g/L (Ref.: 4.53-9.16 g/L), IgA:1.5 g/L (Ref.: 0.2-1 g/L), IgM: 0.82 g/L(Ref.: 0.19-1.46 g/L), however, IVIG therapy was not initiated. Total white blood count was normal at 10.19 x 10^9/L (Ref: 6-13.4 x 10^9/L) with a low-normal absolute lymphocyte count 2.16 x 10^9/L (Ref: 2-7 x 10^9/L); granulocytes normal, 7.2 x 10^9/L (Ref: 1.4-9 x 10^9/L). Lymphocyte subset analysis by flow-cytometry demonstrated 7.4% were CD19 positive (B cells), while CD4 and CD8 T-lymphocytes accounted for 53% and 13% of cells respectively.

Liver function: Transaminases were normal.

P3 – male

3rd child of healthy, Hungarian parents. Sibling, P4, also affected.

Born at 35 weeks gestation, with severe growth retardation, birth weight 990 g, length 38 cm, OFC 28 cm. A diffusely hypertrophic myocardium with mildly dilated right ventricle was noted antenatally. Neonatal respiratory distress treated with surfactant, mechanical ventilation and intravenous antibiotics, (perinatal infection not confirmed), further complicated by a right upper lobe atelectasis on day 8. Neonatal hyperbilirubinemia resolved with phototherapy and anemia treated by red blood cell transfusion (Hgb 78 g/L, Ref: 110-170 g/L).

Developmentally, he had a persisting generalized hypotonia, such that by 1 year he was able to roll over but not able to sit or achieve head control, at which time he was able to smile, and recognize familiar faces. He was attentive and grasped objects. Growth continued to be severely restricted postnatally: at 15 months of age, weight 3000 g, length 55 cm, OFC 38 cm. Feeding was challenging and required occasional nasogastric tube-feeding.

Dysmorphic facial features evident at birth became more prominent with age. There was a relative macrocephaly, prominent occiput, triangular-shaped face with a broad high forehead and frontal bossing. Ears were low-set and very small, the nasal root depressed with a broad nasal base. He had

a long philtrum, horizontal, relatively large mouth with prominent, pouting lips and micrognathia. Blepharophimosis was evident with short palpebral fissures. The hands and feet were small, the backs of the hands and feet were puffy, and the fingers were distally tapering. Patellae were palpable. There was cryptorchism.

Respiratory difficulties persisted postnatally, fluctuating in severity, with tachypnoea, wheeze and bilateral vesicular sounds evident on auscultation. Radiographically, increased right perihilar increased density was frequently evident.

Additionally, recurrent episodes of vomiting ± diarrhea ± fever started at 7 months of age and occurring on a total of nine occasions. At 2 years of age adenovirus infection led to acute respiratory distress syndrome (ARDS) and his death.

There was a persisting mild lymphopenia, lymphocyte absolute count 1.96 x 10^9/L (Ref.: 2.0-7.0 x 10^9/L) and IgA deficiency. IgG 5.38 g/L (Ref.: 5.2-13.6 g/L), IgA: 0.09 g/L (Ref.: 0.36-1.35 g/L), IgM: 1.08 g/L (Ref.: 0.72-1.9 g/L). Intravenous immunoglobulin was administered during one sepsis episode.

At 4 months of age: there was a transient thrombocytopenia of 44 x 10^9/L (Ref.: 150-620 x 10^9/L) in the absence of infection. Platelet transfusion was given during a subsequent episode of thrombocytopenia.

Transaminase levels were elevated: AST 112U/L (Ref: <40 U/L), ALT 64U/L (Ref: <40 U/L), GGT 53 U/L (Ref: <40 U/L) and at 8 months, abdominal ultrasonography revealed a slightly enlarged liver with inhomogeneous structure, with scattered 5-6 mm hypoechogenic islets. Abdominal MRI did not reveal pathological changes in the viscera. Brain MRI was in keeping with 'microcephaly with simplified gyri (MSG)(Adachi et al. 2011), with a medially thin corpus callosum, broad gyri with narrow sulci, and moderately thin cortex reported. Cardiac status improved postnatally, with normal cardiac function and wall-thickness at 20 months of age.

At 7.5 months, he was found to be growth hormone deficient: GH 2.8mg/L (Ref.: <4.7mg/L), IGF-1 9mg/L (Ref.: 33.5-171.8mg/L). and at 9 months, hypothyroidism was detected requiring L-thyroxine treatment: sTSH 11.19 mU/L (Ref.: 0.3-4.2mU/L)(Smith 2007), fT4 and fT3 were normal.

Other investigations. Normal 46 XY karyotype. 4p16.3 FISH normal.

Autopsy: Macronodular hepatic cirrhosis, atrophic large intestine mucosa. In the lung vascular congestion and granular changes in keeping with ARDS. Adrenal hemorrhage, and mild thickening of the left-ventricular wall (7 mm), reflecting a minimal grade myocardial hypertrophy.

P4 -male

Brother of P3, 4th child of Hungarian parents

Born by elective Caesarian section at 38 weeks gestation weighing1640 g, length 40.5 cm, OFC 29 cm. Intrauterine growth restriction evident from 23rd week of the pregnancy. Primordial dwarfism and similarities to deceased brother, including cryptorchidism noted immediately after birth. Neonatally, supplemental nasogastric tube feeding failed to ameliorate failure to thrive. Hypothyroidism diagnosed at 5 weeks, sTSH 11.17 mu/L (Ref.: 6-10 mU/l), fT4, normal: 13.8 pmol/L. Cardiological assessment also identified a 3-4 mm secundum-type atrial defect and mildly dilated right ventricle.

Development was severely delayed, accompanied by generalized muscular hypotonia. Rolling onto to his side was achieved, but no other gross motor milestones were attained. Yet, the patient was attentive, his cognitive status appearing much better than his motor status. Severe growth restriction persisted with extreme microcephalic dwarfism evident at his last assessment at 16 months, weight 2740 g, length 54 cm, OFC 41 cm.

Morphologically, the patient was a phenocopy of his brother: exhibiting relative macrocephaly with frontal bossing, a broad and high forehead, prominent occiput, low-set very small ears, a depressed nasal root, broad nasal base, long philtrum, prominent 'pouting' lips and micrognathia, with limited opening of the mouth. Palpebral fissures were short. The hands and feet were small, the backs of the hands and feet were puffy, with distal tapering of fingers. The patellae were palpable.

From 3 months of age, home oxygen was prescribed because of progressive chronic respiratory difficulties. At 11 months, chest X-ray changes were evident with decreased perihilar and upper-lobe translucency suggestive of pulmonary interstitial disease, along with lower lobe consolidation. From 18 months of age pulmonary hypertension was present and to further clarify the nature of the underlying respiratory disorder, pulmonary biopsy was offered, but declined.

Recurrent episodes of vomiting, diarrhea and a mixed respiratory/metabolic acidosis started from 6 weeks postnatally, presumed infective, although stool cultures were negative. At week 7, rotavirus enteritis caused a life-threatening infection, at week 10, intensive care was needed again following vomiting and diarrhea. Hypogammaglobulinemia prompted initiation of regular IVIG therapy but did not prevent further infections. At 10 weeks of age, IgG: <3g/L (Ref.: 2.32-14.11 g/L), IgA: <0.5g/L (Ref.: 0.36-1.35 g/L), IgM: 0.36g/L (Ref.: 0.72-1.45 g/L). Later on IgA and IgM levels normalized.

Transient thrombocytopenia, at its lowest 20 x 10^9/L, (Ref.: 150-620 x 10^9/L) and anaemia (lowest hemoglobin 78 g/L, Ref.: 90-135 g/L), were corrected with platelet and red blood cell transfusions. A persistent lymphopenia was also present with an absolute lymphocyte count 1.32 x 10^9/L (Ref.: 2.0-7.0 x 10^9/L). Total white blood cell count of 12.07 x 10^9/L, absolute count of 8.31 x 10^9/L (Ref.: 1.4-9.0 x 10^9/L). Flow cytometry demonstrated a profound B cell deficiency with CD19 lymphocytes representing only 0.9% of total; CD3 69%, CD4 43%, CD8 24%, CD56 22%. Phagocyte function: phagocytosis and oxidative burst both normal.

Liver transaminases were deranged: AST 129 U/L (Ref.: <40U/L), ALT 110 U/L (Ref.: <40 U/L), GGT 297 U/L (Ref.: < 40U/L). The liver was palpable 2 cm below the rib-cage appeared inhomogeneous on ultrasound, suggesting a cirrhotic process similar to his brother.

Ophthalmology at 1 year revealed bilateral small central lens opacity.

Death occurred at 19 months of age, thought to be due to rapid cardiorespiratory failure. Autopsy revealed foci of bronchopneumonia and thickened fibrotic alveolar walls. Accompanying this were broadened pulmonary vessel walls and concentric right ventricle hypertrophy, in keeping with pulmonary hypertension. Hepatic fibrosis and a still open anterior fontanelle were also reported.

Individual P5 - male

2nd child born to unrelated British parents. No family history of note

Intrauterine growth retardation was evident during pregnancy, and he was born by emergency Caesarian section at 38 weeks gestation. Birth weight 1.995 kg and OFC 32 cm. At birth, micrognathia, microphthalmia, and cryptorchidism were noted. Neonatal investigations documented bilateral intra-abdominal testes and small kidneys (3 cm length) by ultrasound, while cranial ultrasound,and cardiac echochardiography were normal. A supernumerary ring 20 chromosome (20q11.21-20q13.33) was detected in 3/30 cells from blood, however this was not subsequently detected on array CGH of a skin biopsy, nor by buccal FISH. This was judged insufficient to explain the child's phenotype and trio whole exome sequencing initiated through the DDD study.

Gross motor development was significantly delayed. He rolled at 7 months, and sat unsupported 18/12, contrasting with speech development where first words were evident at 12/12 and 20 words spoken by age 2 years. Vision and hearing were normal. Feeding difficulties were supported by nasogastric feeding and complicated by reflux, however despite these measures weight gain was very poor. Growth continued to be severely restricted, consistent with a severe primordial dwarfism disorder:- at 22 months, weight 3.78 kg, length 55.7 cm, OFC 43.5 cm.

Facially, he had small deep set eyes, short, upslanting palpebral fissures with microphthalmia, micrognathia, and small low set dysplastic ears. Nasal tip was depressed with a smooth philtrum. Small tapering fingers and small nails were also noted, along with eczema. Anterior fontanelle closure was delayed, still 4 cm in width at 2 years of age.

An episode of severe pneumonitis occurred at 4 months of age presumed secondary to aspiration (no organism isolated). Further lower respiratory tract infections were noted at 9 months and at 2 years of age. Radiology; CXR age 1 day for NG tube placement lungs clear, CXR 1 ½ months lungs clear, CXR 4 months widespread pulmonary shadowing which persisted for about 6 weeks - presumed aspiration pneumonitis, CXR by nearly 6 months no consolidation, CXR 9 months patchy bilateral perihilar consolidation consistent with infection, CXR 2 years right perihilar consolidation.

Similar to other cases, there was severe hypogammaglobulinaemia with very low/ undetectable IgG, M, A, E. treated with IVIGs. Consistent with this, B-lymphocytes were absent/ near-absent at 3 months of age, while CD3, CD4, CD8 T-lymphocytes and NK cell numbers were normal. An episode of thrombocytopenia, believed to be sepsis related, resolved spontaneously: Platelets 35 $x10^9$/L at 4 months. This normalized: by 6 months platelets were 406 $x10^9$/L.

Additional investigations demonstrated pancreatic insufficiency. As well alkaline phosphatase at 6 months age was elevated 566 IU/L (normal range 70-250). Renal impairment was also evident with cystatin C level at 21 months 1.25mg/L (normal <1).

He died aged 2 years 3 weeks, as a result of a viral gastrointestinal infection (norovirus detected at Post mortem). Post mortem weight 3090 g, CH length 59.7 cm, CR length 40 cm, OFC 42.5 cm, foot length 7.2 cm. Virtually absent subcutaneous fat, diffuse parenchymal lung disease, non-cirrhotic portal hypertension with regenerative nodular hyperplasia of liver were noted. As well, there was renal hypoplasia, small adrenals with fatty changes, severe atrophy of thymus, an accessory spleen, undescended testes. Post mortem skeletal survey reported thinning of soft tissues around pulp of fingers, bilateral hip dislocation with false acetabulum both sides, gracile bones, widened interpedicular distance in lower lumbar region.

Supplemental References

Adachi Y, Poduri A, Kawaguch A, Yoon G, Salih MA, Yamashita F, Walsh CA, Barkovich AJ. 2011. Congenital microcephaly with a simplified gyral pattern: Associated findings and their significance. *Am J Neuroradiol* **32**: 1123–1129.

Brachmann CB, Davies A, Cost GJ, Caputo E, Li J, Hieter P, Boeke JD. 1998. Designer deletion strains derived from Saccharomyces cerevisiae S288C: A useful set of strains and plasmids for PCR-mediated gene disruption and other applications. *Yeast* **14**: 115–132.

Buchanan BW, Lloyd ME, Engle SM, Rubenstein EM. 2016. Cycloheximide chase analysis of protein degradation in Saccharomyces cerevisiae. *J Vis Exp* **2016**.

Chen W-H, Lu G, Chen X, Zhao X-M, Bork P. 2017. OGEE v2: an update of the online gene essentiality database with special focus on differentially essential genes in human cancer cell lines. *Nucleic Acids Res* **45**: D940–D944.

Danecek P, Auton A, Abecasis G, Albers CA, Banks E, DePristo MA, Handsaker RE, Lunter G, Marth GT, Sherry ST, et al. 2011. The variant call format and VCFtools. *Bioinformatics* **27**: 2156–2158.

Juszkiewicz S, Hegde RS. 2017. Initiation of Quality Control during Poly(A) Translation Requires Site-Specific Ribosome Ubiquitination. *Mol Cell* **65**: 743-750.e4.

Karczewski KJ, Francioli LC, Tiao G, Cummings BB, Alföldi J, Wang Q, Collins RL, Laricchia KM, Ganna A, Birnbaum DP, et al. 2019. Variation across 141,456 human exomes and genomes reveals the spectrum of loss-of-function intolerance across human protein-coding genes. *bioRxiv* 531210.

Larkin MA, Blackshields G, Brown NP, Chenna R, McGettigan PA, McWilliam H, Valentin F, Wallace IM, Wilm A, Lopez R, et al. 2007. Clustal W and Clustal X version 2.0. *Bioinformatics* **23**: 2947–2948.

Laughery MF, Hunter T, Brown A, Hoopes J, Ostbye T, Shumaker T, Wyrick JJ. 2015. New vectors for simple and streamlined CRISPR-Cas9 genome editing in Saccharomyces cerevisiae. *Yeast* **32**: 711–720.

Logan C V., Murray JE, Parry DA, Robertson A, Bellelli R, Tarnauskaitė Ž, Challis R, Cleal L, Borel V, Fluteau A, et al. 2018. DNA Polymerase Epsilon Deficiency Causes IMAGe Syndrome with Variable Immunodeficiency. *Am J Hum Genet* **103**: 1038–1044.

Manichaikul A, Mychaleckyj JC, Rich SS, Daly K, Sale M, Chen W-M. 2010. Robust relationship inference in genome-wide association studies. *Bioinformatics* **26**: 2867–2873.

Niedenthal RK, Riles L, Johnston M, Hegemann JH. 1996. Green fluorescent protein as a marker for gene expression and subcellular localization in budding yeast. *Yeast* **12**: 773–86.

Schrödinger L. 2015. *The PyMOL Molecular Graphics System, Version 2.0*.

Singh G, Cooper TA. 2006. Minigene reporter for identification and analysis of cis elements and trans factors affecting pre-mRNA splicing. *Biotechniques* **41**: 177–181.

Smith L. 2007. Updated AAP guidelines on newborn screening and therapy for congenital hypothyroidism. *Am Fam Physician* **76**: 439.

Spiller MP, Boon K-L, Reijns MAM, Beggs JD. 2007. The Lsm2-8 complex determines nuclear localization of the spliceosomal U6 snRNA. *Nucleic Acids Res* **35**: 923–9.

Van Esch H, Colnaghi R, Freson K, Starokadomskyy P, Zankl A, Backx L, Abramowicz I, Outwin E, Rohena L, Faulkner C, et al. 2019. Defective DNA Polymerase α-Primase Leads to X-Linked

Intellectual Disability Associated with Severe Growth Retardation, Microcephaly, and Hypogonadism. *Am J Hum Genet* **104**: 957–967.

Volland C, Urban-Grimal D, Géraud G, Haguenauer-Tsapis R. 1994. Endocytosis and degradation of the yeast uracil permease under adverse conditions. *J Biol Chem* **269**: 9833–9841.

Waterhouse AM, Procter JB, Martin DMA, Clamp M, Barton GJ. 2009. Jalview Version 2-A multiple sequence alignment editor and analysis workbench. *Bioinformatics* **25**: 1189–1191.
