# Supplementary material for: PRIM1 deficiency causes a distinctive primordial dwarfism syndrome
Source: Genes Dev. 2020 Nov 1;34(21-22):1520–33. doi: 10.1101/gad.340190.120 (PMC7608753; doi:10.1101/gad.340190.120)
Supplement: Supplemental Material [file supp_gad.340190.120_Supplemental_Table_S1.docx]

| **Gene** | **Number of Studies** | **% Studies that Reported Genes as Essential** |
| --- | --- | --- |
| *CDC45* | 9 | 100 |
| *CDC6* | 8 | 100 |
| *CDT1* | 8 | 88 |
| *DONSON* | 9 | 89 |
| *GINS1* | 8 | 88 |
| *MCM4* | 8 | 75 |
| *MCM5* | 9 | 89 |
| *ORC1* | 8 | 88 |
| *ORC4* | 8 | 50 |
| *ORC6* | 8 | 88 |
| *POLA1* | 9 | 89 |
| *POLD1* | 8 | 50 |
| *POLD2* | 8 | 88 |
| *POLE* | 9 | 89 |
| *POLE2* | 9 | 89 |
| *PRIM1* | 7 | 100 |

**Supplemental Table S1: Replisome-associated primordial dwarfism genes are frequently reported as cell essential.** Data from Online GEne Essentiality (OGEE)(Chen et al. 2017).
