# Supplementary material for: PRIM1 deficiency causes a distinctive primordial dwarfism syndrome
Source: Genes Dev. 2020 Nov 1;34(21-22):1520–33. doi: 10.1101/gad.340190.120 (PMC7608753; doi:10.1101/gad.340190.120)
Supplement: Supplemental Material [file supp_gad.340190.120_Supplemental_Table_S2.docx]

| **Gene** | **pLI** | **pRec** |
| --- | --- | --- |
| *CDC45* | 4.62 x 10^-5^ | 1.00 |
| *CDC6* | 1.15 x 10^-9^ | 0.77 |
| *CDT1* | 2.58 x 10^-10^ | 0.27 |
| *DONSON* | 1.35 x 10^-7^ | 0.95 |
| *GINS1* | 7.70 x 10^-7^ | 0.50 |
| *MCM4* | 1.46 x 10^-4^ | 1.00 |
| *MCM5* | 5.93 x 10^-4^ | 1.00 |
| *ORC1* | 1.73 x 10^-14^ | 0.93 |
| *ORC4* | 7.57 x 10^-13^ | 0.23 |
| *ORC6* | 3.36 x 10^-7^ | 0.35 |
| *POLA1** | 1.00 | 7.70 x 10^-10^ |
| *POLD1* | 2.17 x 10^-6^ | 1.00 |
| *POLD2* | 5.64 x 10^-4^ | 0.99 |
| *POLE* | 8.05 x 10^-27^ | 1.00 |
| *POLE2* | 2.85 x 10^-7^ | 1.00 |
| *PRIM1* | 9.94 x 10^-4^ | 1.00 |

**Supplemental Table S2: gnomAD constraint metrics** (Karczewski et al. 2019) **predict primordial dwarfism associated replisome-associated genes to be intolerant to biallelic loss of function.** The probability of loss-of-function intolerance (pLI) score indicates the probability that a gene falls into the category of loss-of-function haploinsufficient genes. pRec, probability that genes are intolerant to biallelic loss-of-function. **POLA1* is located on the X chromosome and the only gene with a greater pLI compared to pRec.
